# MDRSA: A Web Based-Tool for Rapid Identification of Multidrug Resistant *Staphylococcus aureus* Based on Matrix-Assisted Laser Desorption Ionization-Time of Flight Mass Spectrometry

**DOI:** 10.3389/fmicb.2021.766206

**Published:** 2021-12-03

**Authors:** Chia-Ru Chung, Zhuo Wang, Jing-Mei Weng, Hsin-Yao Wang, Li-Ching Wu, Yi-Ju Tseng, Chun-Hsien Chen, Jang-Jih Lu, Jorng-Tzong Horng, Tzong-Yi Lee

**Affiliations:** ^1^Department of Computer Science and Information Engineering, National Central University, Taoyuan, Taiwan; ^2^School of Life and Health Sciences, Warshel Institute for Computational Biology, The Chinese University of Hong Kong, Shenzhen, China; ^3^Department of Laboratory Medicine, Chang Gung Memorial Hospital at Linkou, Taoyuan, Taiwan; ^4^Ph.D. Program in Biomedical Engineering, Chang Gung University, Taoyuan, Taiwan; ^5^Department of Biomedical Sciences and Engineering, National Central University, Taoyuan, Taiwan; ^6^Department of Information Management, National Central University, Taoyuan, Taiwan; ^7^Department of Information Management, Chang Gung University, Taoyuan, Taiwan; ^8^College of Medicine, Chang Gung University, Taoyuan, Taiwan; ^9^Department of Medical Biotechnology and Laboratory Science, Chang Gung University, Taoyuan, Taiwan; ^10^Department of Bioinformatics and Medical Engineering, Asia University, Taichung City, Taiwan

**Keywords:** antibiotics susceptibility test, multidrug resistance, MALDI-TOF MS, machine learning, AST (antibiotic susceptibility testing)

## Abstract

As antibiotics resistance on superbugs has risen, more and more studies have focused on developing rapid antibiotics susceptibility tests (AST). Meanwhile, identification of multiple antibiotics resistance on *Staphylococcus aureus* provides instant information which can assist clinicians in administrating the appropriate prescriptions. In recent years, matrix-assisted laser desorption ionization-time of flight mass spectrometry (MALDI-TOF MS) has emerged as a powerful tool in clinical microbiology laboratories for the rapid identification of bacterial species. Yet, lack of study devoted on providing efficient methods to deal with the MS shifting problem, not to mention to providing tools incorporating the MALDI-TOF MS for the clinical use which deliver the instant administration of antibiotics to the clinicians. In this study, we developed a web tool, MDRSA, for the rapid identification of oxacillin-, clindamycin-, and erythromycin-resistant *Staphylococcus aureus*. Specifically, the kernel density estimation (KDE) was adopted to deal with the peak shifting problem, which is critical to analyze mass spectra data, and machine learning methods, including decision trees, random forests, and support vector machines, which were used to construct the classifiers to identify the antibiotic resistance. The areas under the receiver operating the characteristic curve attained 0.8 on the internal (10-fold cross validation) and external (independent testing) validation. The promising results can provide more confidence to apply these prediction models in the real world. Briefly, this study provides a web-based tool to provide rapid predictions for the resistance of antibiotics on *Staphylococcus aureus* based on the MALDI-TOF MS data. The web tool is available at: http://fdblab.csie.ncu.edu.tw/mdrsa/.

## Introduction

Over the past few decades, inappropriate use of antibiotics has brought out the growth of antibiotic resistance (ABR). More specifically, ABR is the ability of a bacterium to resist the effects of a treated drug and leads to the drug’s ineffectiveness. Using alternative drugs or higher doses of antibiotics to defeat ABR is one of the solutions. However, overusing, underusing, or even misusing the drugs accelerates the growth of ABR. Additionally, it could lead to a bacterium being resistant to a variety of antibiotics, which is knowns as multidrug resistance (MDR), or even called “superbugs.” Meanwhile, superbugs are a huge threat to global health today. One of the well-known superbugs is methicillin-resistant *Staphylococcus aureus* (MRSA), which has become a severe issue all over the world (Wolters et al., 2011; Clerc et al., 2014; Mather et al., 2016).

*Staphylococcus aureus*, a Gram-positive bacterium, is a microorganism commonly found on the skin. These carriers are not symptomatic. However, the pathogen occasionally causes severe diseases including skin, wounds, urinary tract, lung infections, bacteremia, and food poisoning (Naber, 2009). Antibiotics can effectively cure most *Staphylococcus aureus* infections, but MRSA is a bacterium that can resist methicillin and other antibiotics such as oxacillin (OX), penicillin, amoxicillin, and cephalosporin, which are improperly used and produce resistance. It is widely believed that the incorrect use of antibiotics is one of the causes of drug resistance. MRSA has a variety of antibiotic resistance and is generally considered a nosocomial pathogen which causes high mortality (Noskin et al., 2005). Therefore, it is very important to rapidly distinguish between methicillin-sensitive *Staphylococcus aureus* (MSSA) and MRSA.

There are several steps in the current process for determining the treatment of infectious diseases in clinical microbiology. When the doctor suspects that the patient is suffering from a certain infectious disease, the specimens of the infected site are collected for testing. After the specimen collection is completed, the bacterial culture is adopted to provide further bacterial identification. While confirming the bacteria, several antibiotic susceptibility tests (AST) are performed to decide the treatment. In general, it takes about 2–3 days to culture the bacteria and obtain the AST results (Lowy, 2003). Although the standard experiments are highly accurate, the time cost is also high. Before obtaining the AST reports, it is highly dependent on the physicians’ experience to treat patients. Yet, empirical treatments might inadvertently cause more serious drug resistance. In short, the rapid information of AST can reduce ineffective use of drugs.

With the rapid development of antibiotic resistance, several methods for rapid identification of antibiotic resistance have been proposed, such as polymerase chain reaction (PCR) assays and, more recently, the matrix-assisted laser desorption ionization time-of-flight mass spectrometry (MALDI-TOF MS). MALDI-TOF MS is a proteomic tool that measures the molecules including proteins or peptides in the sample. The peptides that are associated with antibiotic resistance might be detected through MALDI-TOF mass spectra. Although qPCR, RT-qPCR, ddPCR, and modified 16S sequencing which obtain AST information in only a few hours could attain high performance, MALDI-TOF MS has more potential to become a convenient and efficient method for identification of antibiotic resistance. The primary reason is that MALDI-TOF MS has already been routinely used in many clinical microbiology laboratories, and there is no additional cost for those that have a MALDI-TOF MS. The mass spectra, generated by MALDI-TOF MS, are composed of peaks of specific mass−to−charge ratios (M/Z) with different intensities, which correspond to a reproducible fingerprint of a certain microorganism (Wang et al., 1998). Consequently, a number of studies have investigated the performance of MALDI-TOF MS on identification of bacterial strains (Ryzhov and Fenselau, 2001; Bizzini et al., 2010; Wang et al., 2018, 2019), and further explored the antibiotics resistance to bacteria (Singhal et al., 2015; Vrioni et al., 2018). Meanwhile, several studies have reported the significant effect on clinical microbiology (Psaroulaki and Chochlakis, 2018; Vrioni et al., 2018; Angeletti and Ciccozzi, 2019; Rodríguez-Sánchez et al., 2019; Welker et al., 2019). In brief, recognizing the pattern of the peptides would serve as a fingerprint for identifying antibiotic resistance in the study, and hence using MALDI-TOF MS to realize the rapid AST in clinical microbiology is promising.

According to the large amount of AST reports collected by Chang Gung Memorial Hospital, the percentages of resistant to erythromycin (E) and clindamycin (CC) were about 50%, which can be seen in Supplementary Figure 1. This implies that providing instant information about the use of them is as critical as the identification of MRSA. However, none of the studies used substantial data or provided a web-based prediction tool for the rapid identifications of oxacillin-, clindamycin-, and erythromycin-resistant *Staphylococcus aureus*. Therefore, the major purpose of this study is to develop a web-based prediction tool, MDRSA, for the rapid identification of multiple drugs resistant to *Staphylococcus aureus* based on a significant amount of MALDI-TOF MS data. Clinicians would obtain instant guidelines about the use of antibiotics for the *Staphylococcus aureus* infection. Additionally, the analysis for the informative peaks would provide more indications for the resistance. In short, development of rapid identification models does contribute an impact on the clinical management of patients with infectious diseases.

## Materials and Methods

### Bacterial Isolates

A total of 20,212 and 5,005 clinical isolates were collected from two medical centers (CGMH Linkou branch and CGMH Kaohsiung branch). These two centers are around 330 km apart. Both centers serve as the referral centers in the regions. It should be noted that these data were collected from the current routine process for determining the treatment of infectious diseases in clinical microbiology. All clinical specimens were collected from all the wards continuously. The specimen types included blood, respiratory tract specimen (sputum, bronchial wash, and bronchoalveolar lavage), sterile cavity fluid (ascites, pleural effusion, pericardial effusion, cerebrospinal fluid, and synovial fluid), urine, and wound. Note that the data collected from Linkou and Kaohsiung branches were regarded as a training set and an independent set, respectively. All the processes of identifying *Staphylococcus aureus* and its resistance strictly followed the Clinical and Laboratory Standard Institute (CLSI) guidelines. Table 1 shows the amount of data in training and independent testing sets. More than 81% of the isolates were recovered from the patient’s sputum, pus, wounds, and blood specimens as shown in Supplementary Table 1.

**TABLE 1:** Number of data in training and independent testing sets.

	Training set		Independent testing set	
Antibiotics	Resistant (%)	Susceptible (%)	Resistant (%)	Susceptible (%)
Oxacillin	10,735 (53.11)	9,477 (46.89)	2,399 (47.93)	2,606 (52.07)
Clindamycin	9,297 (46.00)	10,915 (54.00)	1,880 (37.56)	3,125 (62.44)
Erythromycin	11,304 (55.93)	8,908 (44.07)	2,584 (51.63)	2,421 (48.37)

### Matrix-Assisted Laser Desorption Ionization Time-of-Flight Mass Spectrometry Data Acquisition

MALDI-TOF MS was used to identify the bacterial species and was conducted on Microflex LT (Bruker Daltonik GmbH, Bremen, Germany) benchtop instrument. All isolates were identified as *Staphylococcus aureus* by Bruker MALDI-TOF MS, and the measurement procedures were following the manufacturer’s instructions (Bruker Daltonik GmbH, Bremen, Germany). Mass spectra were acquired in a linear positive mode within a range of +2 kV to +20 Kv and the nitrogen laser frequency was set as 60 Hz.

The species of *Staphylococcus aureus* was analyzed and reported on Biotyper 3.1 software (Bruker Daltonics). Biotyper provided the intensity and the signal quality of the peaks. For each isolate, the maximum number of peaks was set up to 200 and the acceptable quality is larger 2.0 which is the benchmark from the instruction of Biotyper 3.1. Furthermore, by using Flexanalysis 3.4 (Bruker Daltonik GmbH, Bremen, Germany) we could get a mass list, the parameters were set as follows: centroid peak detection algorithm for peak finding; Top Hat method for baseline subtraction; signal-to-noise threshold was set as 2; the minimum peak width expected in the spectrum was set as 6 M/Z; the maximal number of peaks was set as 200; relative intensity threshold was set as 0%; minimum intensity threshold was set as 0, and height was set as 80%. In this investigation, spectra ranging from 2,000 to 20,000 M/Z were acquired for further analysis.

### Spectral Data Processing

Even in the same experimental steps and environment, the MALDI-TOF mass spectra of the same isolates may still be different. Specifically, the strong peaks on different MALDI-TOF mass spectra of the same strain may not be located at the same M/Z (Lin et al., 2005; AlMasoud et al., 2014), and we called this problem a shifting problem. Consequently, preprocessing for each single mass spectrum before constructing the models is an essential step, especially for large-scale data derived from the clinical medicine.

In order to deal with the peak shifting problem that appears in MALDI-TOF MS data, the kernel density estimation (KDE) was adopted to estimate the actual location of the peaks. Specifically, KDE is a non-parametric method to estimate the probability density function (PDF) of a random variable (Sheather and Jones, 1991). The M/Z values were regarded as the random variable. We then applied the KDE with Gaussian kernel to estimate the PDF of the M/Z values, which can be represented as

(1) $$f\,\left(x\right)=\frac{1}{n\,h}\,\sum_{i=1}^{n}K\,\left(\frac{x-x_{i}}{h}\right)=\frac{1}{\sqrt{2\,\pi }\,n\,h}\,\sum_{i=1}^{n}\exp\left\{-\frac{1}{2}\,{\left(\frac{x-x_{i}}{h}\right)}^{2}\right\}$$

where *x*_1_, *x*_2_, …, *x*_*n*_ are all M/Z values derived from all spectra, *h* is the bandwidth, which is also the smoothing parameter, *n* is the number of M/Z values, and *K* is the kernel function.

**FIGURE 1:**
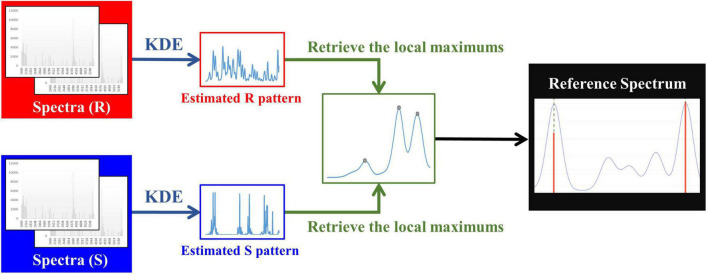
Flow chart of constructing a reference spectrum template.

To obtain M/Z patterns for resistant and susceptible spectra, we used the function “stats.gaussian_kde,” provided by SciPy (Virtanen et al., 2020), to estimate their PDFs in this study. It should be noted that the bandwidth is a critical parameter for employing KDE. The parameter “bw_method” provided in “stats.gaussian_kde” can be used to determine it. More specifically, if “bw_method” is a scalar, the bandwidth will be the scalar multiplied by the standard deviation of the sample. After the PDFs of M/Z patterns for resistant and susceptible spectra were obtained, the local modes derived from two PDFs were retrieved and concatenated to be a one spectrum with several peaks. Then we removed the duplicate values to construct a reference spectrum template. In addition to removing the duplicate values, the distance between two adjacent local modes less than three were also removed. The minimum width of two adjacent peaks expected in a spectrum was set as 6 M/Z in Flexanalysis 3.4. Finally, these M/Z values formed the final reference spectrum template. Figure 1 demonstrates the flow chart of constructing a reference spectrum template. Note that 0.0006, 0.0008, 0.001, 0.0012, and 0.0014 were the values of “bw_method” in this study and used to generate different reference spectra. Their corresponding bandwidths were 2.08, 2.77, 3.46, 4.15, and 4.84. The peaks in every spectrum were then aligned to the nearest ones in the reference spectrum accordingly. Supplementary Figure 2 illustrates the alignment for mass spectrum of Isolate A. More specifically, the purple line is the PDF of the population, so all the peaks of a mass spectrum should be shifted to the nearest benchmark which is the local maximum of PDF. Supplementary Table 2 all features (peaks) used for developing oxacillin (OX), clindamycin (CC), and erythromycin (E) models.

The amount of the intensity may be influenced by various factors like temperature, instrument set-up, storage, and manual operation (Baumann et al., 2005), so we need to process the raw spectra data first. In this study, we scaled each intensity by its spectrum’s maximum intensity. The definition of the formula is given below:

(2) $$y_{i\,j}^{{}^{*}}=\frac{y_{i\,j}}{\max\left\{y_{i\,j}|i=1,2,\ldots ,n_{j}\right\}}$$

where $y_{i\,j}^{{}^{*}}$ and *y*_*ij*_ are the scaled and original intensities for the *j*th spectrum at the *i*th peak, respectively, and max (*y*_*ij*_| *i* = 1, 2, …, *n*_*j*_) is the maximum intensity for the *j*th spectrum which contains *n*_*j*_ peaks.

### Model Construction

After preprocessing the MS data, we adopted three machine learning (ML) algorithms, including decision tree (DT), random forest (RF), and support vector machine (SVM), to build up the classification models to predict the antibiotic resistance. Further information about the algorithms was then given in the next paragraph. The grid search with 10-fold cross validation was implemented on the training set for each bandwidth. When the optimal parameters were obtained, the independent testing set was used to evaluate the performance based on the model trained by the whole training set.

DT is a commonly used method for building the classification models. DT is formed in a tree-like structure which is constructed by nodes and leaves. Each node represents a test on a feature and each branch stands for an outcome of the test. Lastly, each leaf represents the class resulting from all tests. The criterion for yielding the best classification is important. Classification and regression trees (CART) algorithm is one of the commonly used algorithms to produce the best classification. The CART algorithm is a greedy approach that allows each step to select an optimal feature to get the most information gain when selecting attributes (Breiman et al., 1984). The measurement for selecting the optimal feature is finding the minimum impurity. In this study, we used the Gini index as the approach for calculating the impurity, which is the most common assessment approach. For each selection, the sum of the Gini impurity for all branches will be calculated, and the minimum one will be the best selection. The function “sklearn.tree.DecisionTreeClassifier” in scikit-learn package was used to build the DT model (Pedregosa et al., 2011).

RF is another common machine learning classifier, composed of multiple optimized version of CARTs to build the prediction model. RF uses bootstrap aggregating (bagging), one of the ensemble learning methods, to make sure each tree randomly gets training sets and attributes. As illustrated in Supplementary Figure 3, the ensemble learning method trains multiple models and votes the result finally, and the data used in each model was randomly determined in reusable. The classification outcome of RF is determined by the mode of every individual tree output. Most of the time, compared to DT, RF performs well when dealing with many features. Other reasons we use RF are that the learning time is short, and it can assess the importance of features easily. In this research, the tool we used to build the RF classification model is the function “sklearn.ensemble.RandomForestClassifier” in scikit-learn package (Pedregosa et al., 2011).

Support vector machine (SVM) is another common supervised learning classification. SVM finds a hyperplane that can minimize the risk of misclassification. The method used to minimize the risk is to find a decision boundary that can maximize the boundaries between the two classes. As shown in Supplementary Figure 4, there are two classes on a plane. We can find many possible hyperplanes that can separate two classes, and the algorithm for SVM is to find the hyperplane that can “maximum” the distance (the largest margin) between two classes. In this study, we use the function “sklearn.linear_model.SGDClassifier” in scikit-learn package (Pedregosa et al., 2011).

### Statistical Analysis

Chi-squared test and *t*-test were employed in this study to evaluate the capability of discriminating the resistance for an individual peak based on their presence and intensities, respectively. Specifically, the chi-squared test of independence was mainly conducted to test the correlations between two categorical variables. In short, the small *p*-values concluded that the presence of a specific peak was correlated to the resistance. On the other hand, *t*-test was used to compare the intensities between two groups. Similarly, the small *p*-value would refer to that the intensity of a specific peak was different between two groups.

### Evaluation Metrics

In this study, we used accuracy (ACC), the area under the receiver operating characteristic curve (AUC), sensitivity (SN), very major error (VME), specificity (SP), major error (ME), and Matthew’s correlation coefficient (MCC) as the performance measurements for our models. The definitions of these measurements are given below.

(3) $$A\,C\,C=\frac{T\,P+T\,N}{T\,P+T\,N+F\,P+F\,N}$$

(4) $$S\,N=\frac{T\,P}{T\,P+F\,N}$$

(5) $$V\,M\,E=\frac{F\,N}{T\,P+F\,N}$$

(6) $$S\,P=\frac{T\,N}{F\,P+T\,N}$$

(7) $$M\,E=\frac{F\,P}{F\,P+T\,N}$$

(8) $$M\,C\,C=\frac{T\,P\times T\,N-F\,P\times F\,N}{\sqrt{\left(T\,P+F\,P\right)\,\left(T\,P+F\,N\right)\,\left(T\,N+F\,P\right)\,\left(T\,N+F\,N\right)}},$$

where TP is true positive which means the number of antibiotic-resistant isolates are correctly predicted by the classifier, TN is true negative which means the number of antibiotic-sensitive isolates are correctly predicted by the classifier, FP is false positive which means the number of antibiotic-sensitive isolates are wrongly predicted as antibiotic-resistant isolates by the classifier, and TN is false negative which means the number of antibiotic-resistant isolates are wrongly predicted as antibiotic-sensitive isolates by the classifier. Accuracy is the rate of the difference between the prediction results and the real results. MCC is the measurement to measure the quality of the binary classification. It returns a value between −1 and +1. If MCC returns +1, it means the prediction is perfect; if MCC is 0, if MCC returns −1, it represents the prediction is totally wrong. MCC considers the case that the sizes of the classes are very different and gives a balanced measurement.

In medicine, it is often determined by some thresholds whether the prediction result is true or false, and this threshold will affect the sensitivity and the specificity. In short, different threshold sets will lead to different prediction results. The distribution of the different threshold sensitivity and specificity can be plotted as the ROC curve, and the area under the ROC curve is called AUC. The most ideal case is AUC = 1, which is the case that the point locates on the upper left corner of the plot; when the AUC is 0.5, it represents a random selection of conditions, which means random guess. Most cases are within these two values. Through ROC and AUC, we can choose a more robust and stable model.

### Development of a Web-Based Prediction Tool

We used hypertext markup language (HTML) and hypertext preprocessor (PHP) with python code to implement a web-based prediction tool in the backend upon submission of MALDI-TOF MS data. Each MS data should start with “BEGIN IONS” and end with “END IONS.” This web-based prediction tool could predict one or more MS data for a submission. This web-prediction tool would list the prediction probabilities for the submitted MS data show the submitted MS figure with the important features.

## Results

### MS Data Overview

**FIGURE 2:**
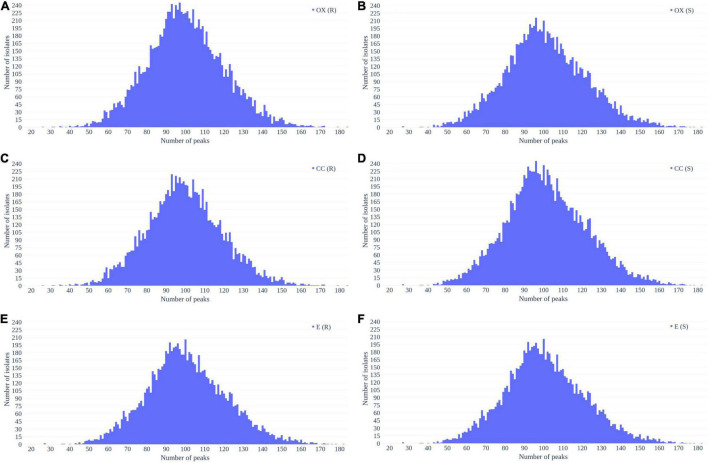
Distribution of number of peaks retrieved from each spectrum in **(A)** oxacillin-resistant, **(B)** oxacillin-susceptible, **(C)** clindamycin-resistant, **(D)** clindamycin-susceptible, **(E)** erythromycin-resistant, and **(F)** erythromycin-susceptible *Staphylococcus aureus*.

Figure 2 shows the number of peaks in each spectrum according to different antibiotics resistance. Most of spectra preserved 50–150 peaks. Since most of the data were overlapped, there is no significant difference between the number of peaks between resistant and susceptible strains.

**FIGURE 3:**
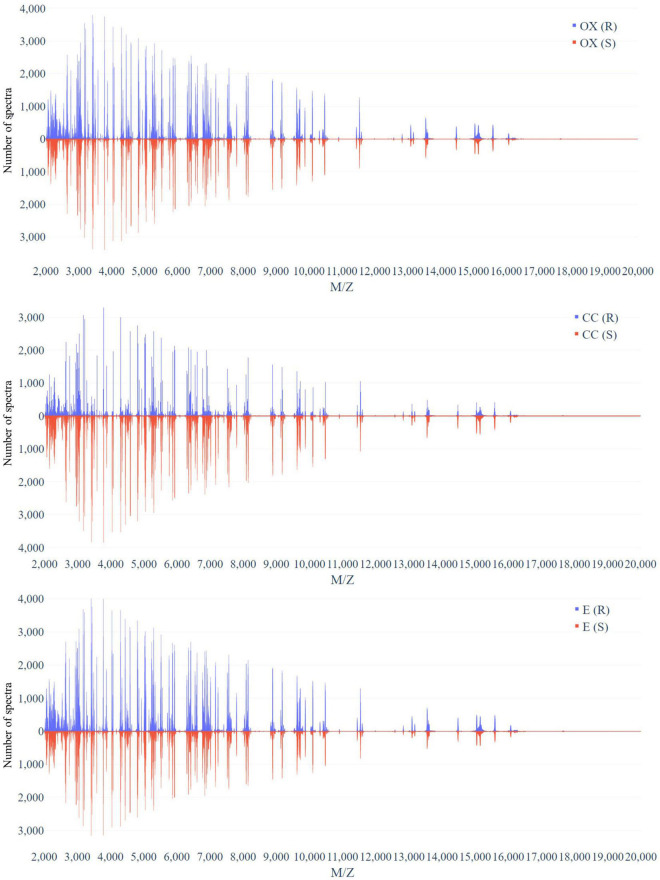
Distribution of number of spectra that were derived from oxacillin- (upper), clindamycin- (middle), and erythromycin-resistant/susceptible (bottom) *Staphylococcus aureus* isolates at each M/Z.

**FIGURE 4:**
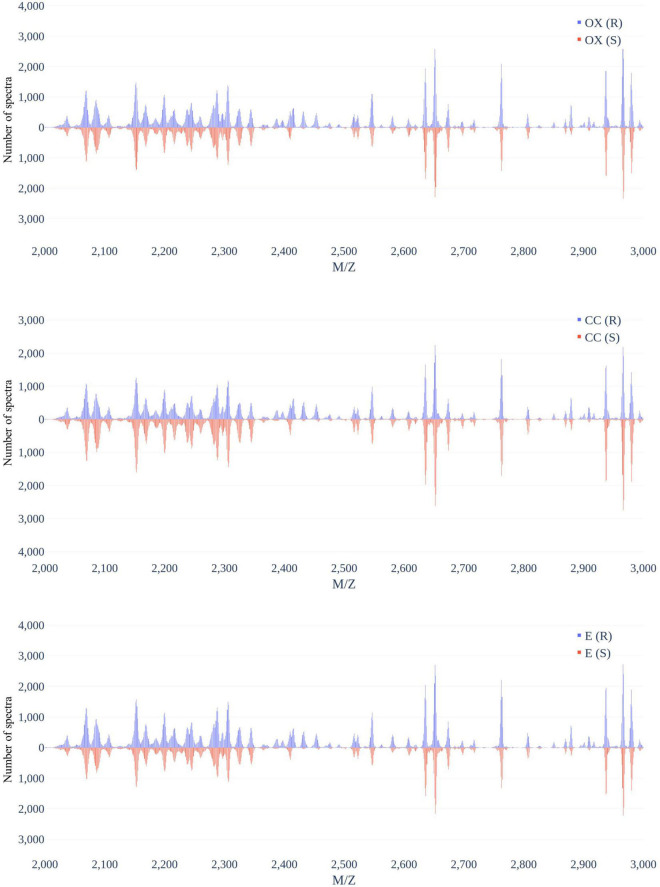
Distribution of number of spectra that were derived from oxacillin- (upper), clindamycin- (middle), and erythromycin-resistant/susceptible (bottom) *Staphylococcus aureus* isolates at M/Z = 2,000–3,000.

Figure 3 demonstrates the distribution of the number of spectra that were derived from oxacillin-, clindamycin-, and erythromycin-resistant/susceptible *Staphylococcus aureus* isolates at M/Z = 2,000–20,000. Since the range of M/Z is too large to obtain detailed information, we then further zoomed in to the M/Z range 2,000–3,000 to find some information (Figure 4). Peaks at M/Z = 2,360–2,500 are different between resistant and susceptible strains for all three antibiotics. We summed all intensities of resistant and susceptible isolates to observe the difference between them. It was still difficult to compare the difference between resistance and susceptibility (Supplementary Figure 5), so we zoomed in on these figures to find the differences. We found that it still has the difference of resistance and susceptibility at the range from 2,360 to 2,500 M/Z for oxacillin, clindamycin, and erythromycin (Supplementary Figure 6).

### Performance of Prediction Models

To build up a stable model, the parameters of the model are critical, especially the bandwidth. The bandwidth of Gaussian KDE is crucial. If the bandwidth is too large, the PDF will be too smooth; if the bandwidth is too small, it will be too harsh. We adopted 10-fold cross-validation models with different “bw_method” parameters and different ML algorithms to find the optimal bandwidth parameter. We tried the “bw_method” parameters from larger to smaller, and we found that when the “bw_method” parameter approaches 0.001, the accuracy tends to be stable. Supplementary Table 3 shows the results of 10-fold cross-validation with the optimal parameters based on the grid search with different “bw_method” parameters of Gaussian KDE on the training set of oxacillin, clindamycin, and erythromycin, respectively. We could find that when the “bw_method” parameter was set to 0.0008, the standard deviations of oxacillin and clindamycin models were small and retained high accuracy Similarly, E model would reach the optimal when the bw_method is 0.001. Moreover, the highest accuracies are all built by the RF algorithm.

We adopted the optimal parameters to construct the RF-based models for the three antibiotics based on the whole training set. These models were then tested by the independent testing set and compared with those that did not use the KDE preprocessing. When the KDE preprocessing was adopted, the accuracies were 81.42, 82.20, and 74.63% for oxacillin, clindamycin, and erythromycin, respectively (Table 2). Comparing to the models that used data without KDE preprocessing, the accuracies derived from KDE were higher (6.04, 5.78, and 6.58%) for oxacillin, clindamycin, and erythromycin, respectively.

**TABLE 2:** Results of with or without kernel density estimation (KDE) preprocessing on independent testing set.

Antibiotics	Metrics	Without KDE preprocessing	Using KDE preprocessing
OX	SN	0.7962	0.7524
	VME	0.2038	0.2476
	SP	0.7149	0.8711
	ME	0.2851	0.1289
	ACC	0.7538	0.8142
	AUC	0.7555	0.8117
CC	SN	0.7282	0.6489
	VME	0.2718	0.3511
	SP	0.7859	0.9261
	ME	0.2141	0.0739
	ACC	0.7642	0.8220
	AUC	0.7571	0.7875
E	SN	0.7693	0.6908
	VME	0.2307	0.3092
	SP	0.5857	0.8055
	ME	0.4143	0.1945
	ACC	0.6805	0.7463
	AUC	0.6775	0.7481

*OX, oxacillin; CC, clindamycin; E, erythromycin; SN, sensitivity; VME, very major error; SP, specificity; ME, major error; ACC, accuracy; AUC, area under the receiver operating characteristic curve.*

### Forward Feature Selection

To obtain a more informative feature set, the feature importance scores calculated by RF was determined in this study. More specifically, we used the 70% training set to build up the classification models and calculated the features’ importance scores based on the RF algorithm. The features were then ranked by their importance scores. After that, the feature was added in the model sequentially until the accuracy of the remaining 30% training set reached a plateau. Supplementary Figure 7A shows the trend of accuracy as the feature was added sequentially for the oxacillin model. When the number of features is 36, the model reaches a plateau and accuracy is 84.13%. The clindamycin model, demonstrated in Supplementary Figure 7B, attained a plateau at 37 features with an accuracy of 80.22%. Thirty-seven features were used to reach a plateau for the erythromycin model as shown in Supplementary Figure 7C. Table 3 shows the performance of the selected features on the independent testing set. When the number of features reduced to about 40, the accuracy was still around 80%. Furthermore, 589, 600, and 824 data were incorrectly called as sensitive for oxacillin, clindamycin, and erythromycin models, respectively. Meanwhile, 384, 280, and 442 data were incorrectly called as resistant for oxacillin, clindamycin, and erythromycin model, respectively.

**TABLE 3:** Performance of features selection on independent test set.

	Model		
	Oxacillin	Clindamycin	Erythromycin
Number of features	36	38	37
Sensitivity	0.7545	0.6809	0.6811
Very major error	0.2455	0.3191	0.3189
Specificity	0.8526	0.9104	0.8174
Major error	0.1474	0.0896	0.1826
Accuracy	0.8706	0.8242	0.7471
AUC	0.8036	0.7956	0.7493

*AUC, Area under the receiver operating characteristic curve.*

Supplementary Table 4 lists all selected features for each model. We found most of the selected peaks were duplicated, but some peaks were selected uniquely for a certain model. More specifically, the peaks at 11,539, 4,526, and 3,297 M/Z were only selected by the oxacillin model. While the peaks at 2,910, 3,045, 2,966, and 7,568 M/Z were only included by the clindamycin model after the feature selection. The erythromycin model incorporated peaks at 6,524, 4,514, 5,004, and 2,652 M/Z, which were not selected by other models. In addition, the peak at 6,593 M/Z ranked first for the oxacillin and erythromycin models. But the clindamycin model ranked it at a 14th place. This implies that the characteristics of resistance to clindamycin would be different from oxacillin and erythromycin.

In order to further investigate the selected peaks, we used the chi-square test for comparing two proportions of the resistant and susceptible data. Additionally, we also employed the *t*-test for comparing the intensities for these two groups. The results of these two statistical tests are shown in Supplementary Tables 5–7 for the oxacillin model, the clindamycin model, and the erythromycin model, respectively. In this study, the *p*-value less than 0.001 was claimed as statistically significant. Most *p*-values of chi-square tests for the selected peaks were shown the significant difference between resistant and susceptible data. Yet, some selected peaks did not indicate the statistical significance such as peaks at 6,553, 5,526, and 3,277 M/Z for the clindamycin model when the chi-square test was adopted (Supplementary Table 5). While the *t*-test was employed to compare two intensities, several peaks did not show the significant difference such as the peaks at 3,008, 3,045, 2,200, 6,424, 6,890, and 2,966 M/Z for the clindamycin model and the peaks at 6,553, 2,306, 3,056, 2,287, and 7,021 M/Z for the erythromycin model.

**FIGURE 5:**
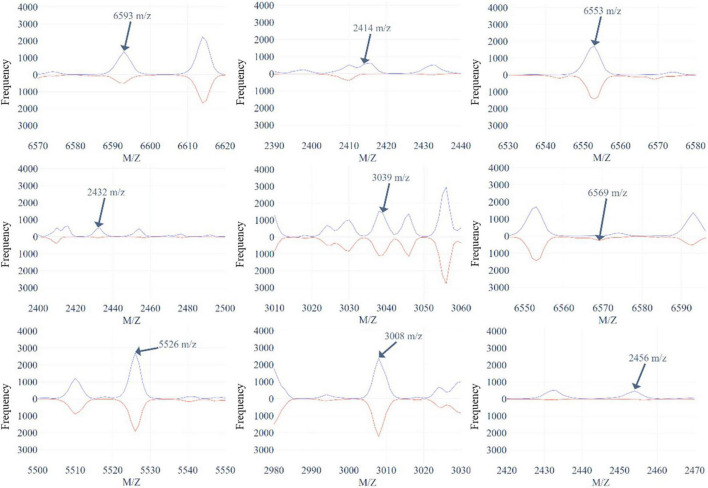
The top 9 selected peaks distributions of the M/Z values without peaks alignment for oxacillin-resistant (red)/susceptible (blue) data.

Figure 5 and Supplementary Figures 8, 9 demonstrate the top 9 selected peak distributions of the M/Z values without peak alignment for three models to further investigate the difference on oxacillin-, clindamycin-, and erythromycin-resistant/susceptible data, respectively. These figures also indicate that the resistant isolates have more chance to appear at some specific peaks than the susceptible ones such as peaks at 6,593, 2,414, 2,432, and 2,456 M/Z on oxacillin data; peaks at 2,414, 2,432, 2,456, and 7,595 M/Z on clindamycin data; and peaks at 6,593, 2,413, 2,432, and 2,456 M/Z on erythromycin data.

### Investigation of Multidrug Resistance

A Venn diagram was used to demonstrate the multiple antibiotics resistance, which is shown in Supplementary Figure 10. About 41% (8,234/20,212) of isolates were resistant to three antibiotics, and 37% (7,455/20,212) of isolates were susceptible to three antibiotics. This implies that most of the isolates were either resistant to three antibiotics or susceptible to them. Due to the few numbers of only resistant to a specific antibiotic or two antibiotics, we constructed a binary classification model to discriminate that the isolate is resistant or susceptible to three antibiotics simultaneously. Supplementary Table 8 shows the amount of data for this classification. Similarly, we used 10-fold cross validation to find the best parameters and models. The performance is shown in Supplementary Table 9. The best AUC obtained from the RF model had a bandwidth of 0.0006. According to the optimal parameters derived from the training set, the performances on the independent testing set were 0.7918 (sensitivity), 0.9053 (specificity), 0.8545 (accuracy), 0.7057 (MCC), and 0.8486 (AUC).

### RDMDRSA Web Interface

Based on our method, an online prediction server—MDRSA—was developed to predict the possibility that an MS derived from a *Staphylococcus aureus* isolate might be resistant to a particular antibiotic. The best prediction models developed for identifying the oxacillin-, clindamycin-, and erythromycin-resistant were applied here. Screenshots of the website are shown in Supplementary Figure 11.

## Discussion and Conclusion

In this study, we used the MALDI-TOF MS data from Chang Gung Memorial Hospital Linkou branch to build different ML models to identify the resistance of the *Staphylococcus aureus*, and the data from Chang Gung Memorial Hospital Kaohsiung branch were further adopted to evaluate these models. Additionally, we adopted the Gaussian KDE method to deal with the shifting problem in MS data. Note that the bandwidth selection was based on the mean accuracy of the 10-fold cross validation. The accuracies of the 10-fold cross validation models were 86.28, 85.66, and 80.93% for oxacillin, clindamycin, and erythromycin models, respectively. Meanwhile, the accuracies of the independent testing were attained to 81.42, 82.20, and 74.63% for oxacillin, clindamycin, and erythromycin models, respectively. The forward feature selection was further used to reduce the dimension of features according to the order of importance derived from the RF. We then selected 36, 38, and 37 features for oxacillin, clindamycin, and erythromycin models, respectively. The accuracies of the models used selected features on the independent testing set were 80.56, 82.42, and 74.71% for oxacillin, clindamycin, and erythromycin models, respectively. The investigation of multiple drug resistance demonstrated that most isolates were either resistant to three antibiotics or susceptible to them. The accuracy of independent testing was 85.46% which was higher than the models that were used for identifying a specific resistance.

Previous studies were mainly devoted to identifying methicillin-resistant *Staphylococcus aureus* (MRSA), and figuring out their informative peaks (Wang et al., 2013, 2020; Josten et al., 2014; Østergaard et al., 2015; Camoez et al., 2016; Rhoads et al., 2016; Bai et al., 2017; Sogawa et al., 2017; Kim et al., 2019; Tang et al., 2019; Liu et al., 2021). Bai et al. (2017) proposed a genetic algorithm with a *t*-test based population seeding for wrapper feature selection on 727 *Staphylococcus aureus* clinical isolates’ mass spectra derived from Vitek MS, and their accuracy based on support vector machine classifier was 0.72. Sogawa et al. (2017) utilized support vector machine to discriminate MRSA from methicillin-susceptible *Staphylococcus aureus* (MSSA) based on features derived from MALDI-TOF mass spectra. Their model reached prediction accuracies of over 85% and significantly reduced the time to initiation of targeted antibiotic treatment in comparison with phenotypic resistance profiling. Yet, they only considered 160 clinical isolates. Kim et al. (2019) developed discrimination models based on 320 clinical *Staphylococcus aureus* clinical isolates’ mass spectra and 181 new ones were tested, and the DT had a sensitivity of 87.6%. Tang et al. (2019) applied different supervised ML models which are capable of distinguishing MRSA from MSSA. Even though their prediction accuracy was over 90%, only 20 isolates were used. Liu et al. (2021) used R to analyze 452 *Staphylococcus aureus* clinical isolates’ mass spectra derived from Vitek MS, and the best area under the receiver operating characteristic curve was 0.89 by support vector machine. Compared with previous studies, our study used much clinical data and considered three antibiotics.

The limitation for analyzing antibiotic resistance through MALDI-TOF MS is that some antibiotic resistance-related peptides might not be detectable through mass spectra derived from MALDI-TOF MS when using the routine sample preparation protocol. This would limit the prediction for the antibiotic resistance. Yet, we incorporated data from two medical centers which are around 330 km apart. Given the spatial distribution of the two medical centers, we would detect the spectral pattern that is associated with antibiotic resistance. However, the possibility of detecting specific clones could not be fully excluded now without molecular strain typing data. Moreover, some factors including culture medium, bacteria lysis condition, and matrix crystallization condition, would have impact on the MALDI-TOF mass spectra and the subsequent identification of antibiotic resistance. Meanwhile, bacterial strains in different regions are quite diverse. Although it would be unsuitable to apply our models in other regions, we proposed a valid method to deal with the peak-shifting problem of MALDI-TOF MS. Specifically, the local MS data needed to be collected and our methods employed to develop the proper prediction models. On the other hand, our MALDI-TOF MS data were obtained from Bruker Daltonics GmbH. We did not compare with different MS data which was derived from different systems in the study. In addition, we did not further identify the proteins for the informative peaks. Even so, the results did show that the proportions of resistant were higher than the non-resistant ones for the selected peaks. The further identification of the informative peaks could provide a more comprehensive view on the mechanism of antibiotic resistance and would be valuable for the development of potential new treatments.

In this study, both accuracy and AUC for the internal (10-fold cross validation) and external (independent testing) validation attained 0.8. The promising results can provide more confidence to apply these prediction models in the real world. Briefly, this study provides a web-based tool to provide rapid predictions for the resistance of antibiotics on *Staphylococcus aureus* based on the MALDI-TOF MS data. In the future, a cross-national study is required. Given the high diversity of microorganisms across countries, it is not possible that the current prediction models can be used in other areas/countries without adjustment. Training and validating machine learning models based on locally relevant MALDI-TOF MS data are favorable.

## Data Availability Statement

The raw data supporting the conclusions of this article will be made available by the authors, without undue reservation.

## Author Contributions

C-RC, J-MW, and H-YW carried out the data collection and curation. C-RC, ZW, and J-MW participated in the data analyses, model construction, and drafted the manuscript. C-RC, ZW, J-MW, H-YW, L-CW, and T-YL participated in the design of the study and performed the draft revision. J-TH, Y-JT, C-HC, T-YL, and J-JL conceived of the study, participated in its design and coordination, and helped to revise the manuscript. All authors read and approved the final manuscript.

## Conflict of Interest

The authors declare that the research was conducted in the absence of any commercial or financial relationships that could be construed as a potential conflict of interest.

## Publisher’s Note

All claims expressed in this article are solely those of the authors and do not necessarily represent those of their affiliated organizations, or those of the publisher, the editors and the reviewers. Any product that may be evaluated in this article, or claim that may be made by its manufacturer, is not guaranteed or endorsed by the publisher.
