# Supplementary material for: MDRSA: A Web Based-Tool for Rapid Identification of Multidrug Resistant Staphylococcus aureus Based on Matrix-Assisted Laser Desorption Ionization-Time of Flight Mass Spectrometry
Source: Front Microbiol. 2021 Dec 3;12:766206. doi: 10.3389/fmicb.2021.766206 (PMC8678511; doi:10.3389/fmicb.2021.766206)
Supplement: Supplementary file 1 [file Data_Sheet_1.pdf]

MDRSA: a web based-tool for rapid identification of multidrug resistant *Staphylococcus aureus* based on matrix-assisted laser desorption ionization-time of flight mass spectrometry

Chia-Ru Chung[1,†], Zhuo Wang[2,†], Jing-Mei Weng[1], Hsin-Yao Wang[3,4], Li-Ching Wu[5], Yi-Ju Tseng[3,6], Chun-Hsien Chen[3,7] , Jang-Jih Lu[3,8,9,*], Jorng-Tzong Horng[1,10,*], and Tzong-Yi Lee[2,*]

[1]Department of Computer Science and Information Engineering, National Central University, Taoyuan, Taiwan
[2]Warshel Institute for Computational Biology, School of Life and Health Sciences, The Chinese University of Hong Kong, Shenzhen, China
[3]Department of Laboratory Medicine, Chang Gung Memorial Hospital at Linkou, Taoyuan, Taiwan
[4]Ph.D. Program in Biomedical Engineering, Chang Gung University, Taoyuan, Taiwan
[5]Department of Biomedical Sciences and Engineering, National Central University, Taoyuan 32001, Taiwan
[6]Department of Information Management, National Central University, Taoyuan, Taiwan
[7]Department of Information Management, Chang Gung University, Taoyuan, Taiwan
[8]College of Medicine, Chang Gung University, Taoyuan, Taiwan
[9]Department of Medical Biotechnology and Laboratory Science, Chang Gung University, Taoyuan, Taiwan
[10]Department of Bioinformatics and Medical Engineering, Asia University, Taichung, Taiwan

[†]These authors contributed equally to this work.
[*]To whom correspondence should be addressed: JJ Lu: janglu45@gmail.com, JT Horng: horng@db.csie.ncu.edu.tw and TY Lee: leetzongyi@cuhk.edu.cn

Keywords: antibiotics susceptibility test, multidrug resistance, MALDI-TOF MS, machine learning

Supplementary Information

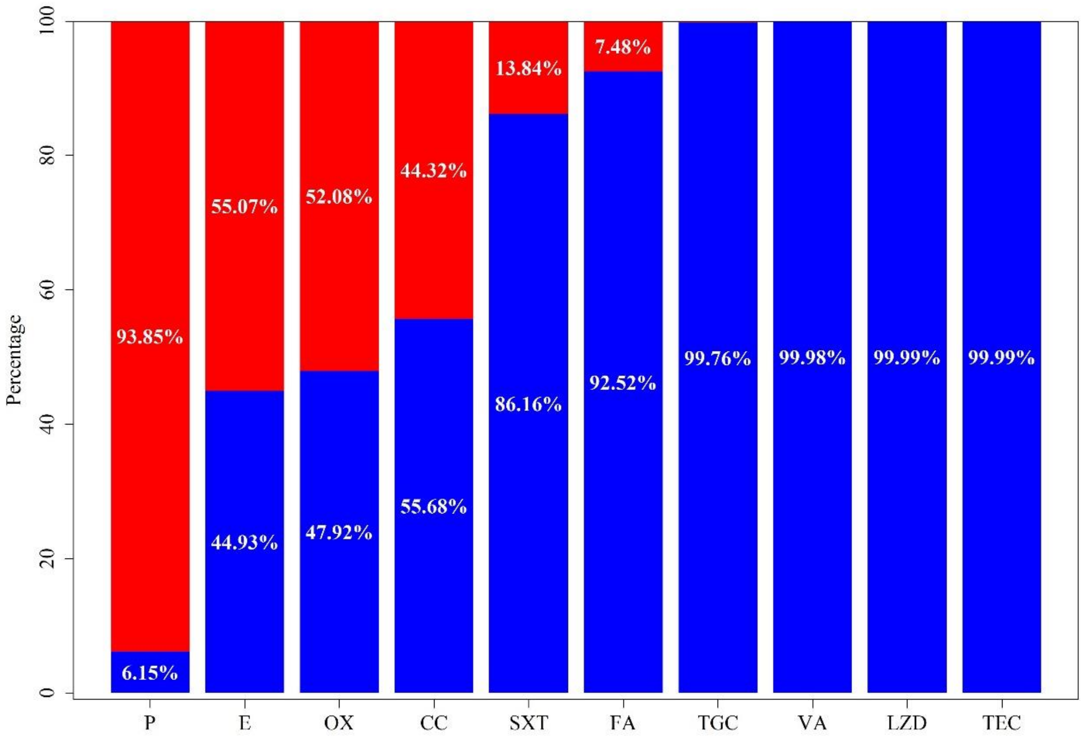

Supplementary Figure 1. Percentages of susceptible (blue) and resistant (red) *Staphylococcus aureus* isolates for penicillin (P), erythromycin (E), oxacillin (OX), clindamycin (CC), sulfamethoxazole-trimethoprim (SXT), fusidic acid (FA), tigecyclin (TGC), vancomycin (VA), linezolid (LZD), and teicoplanin (TEC).

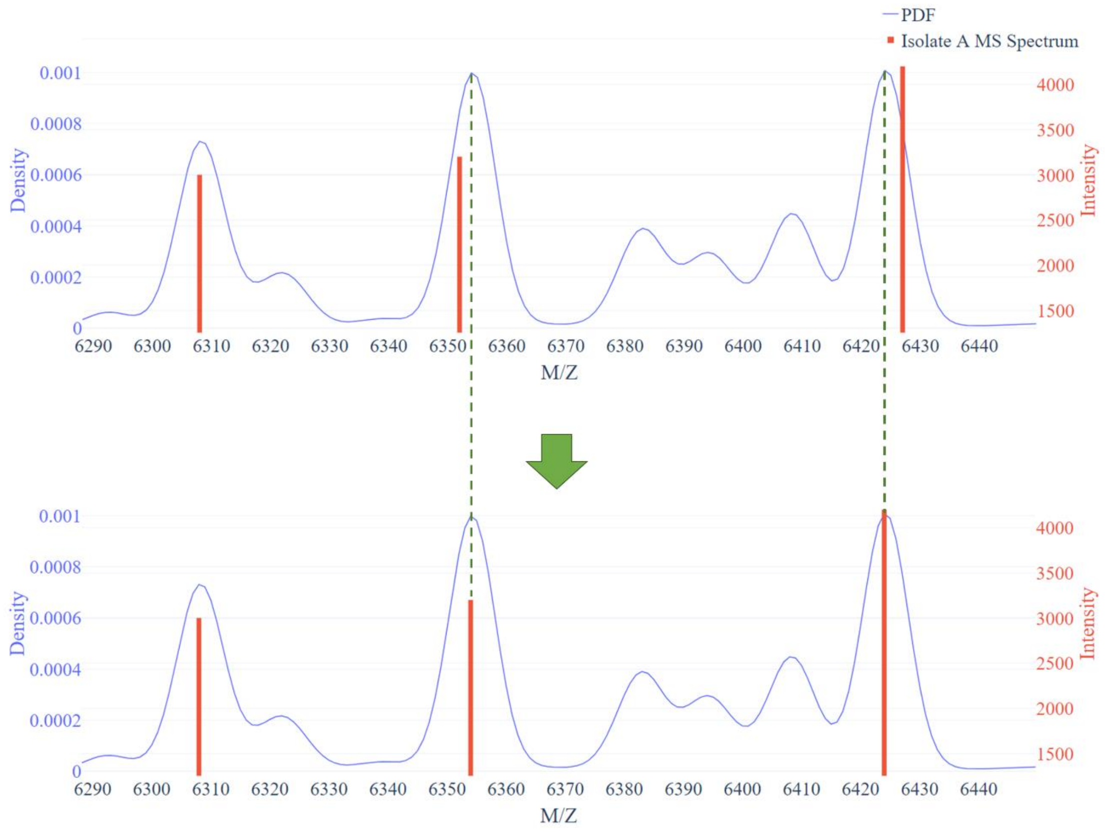

Supplementary Figure 2. Illustration of peak alignment.

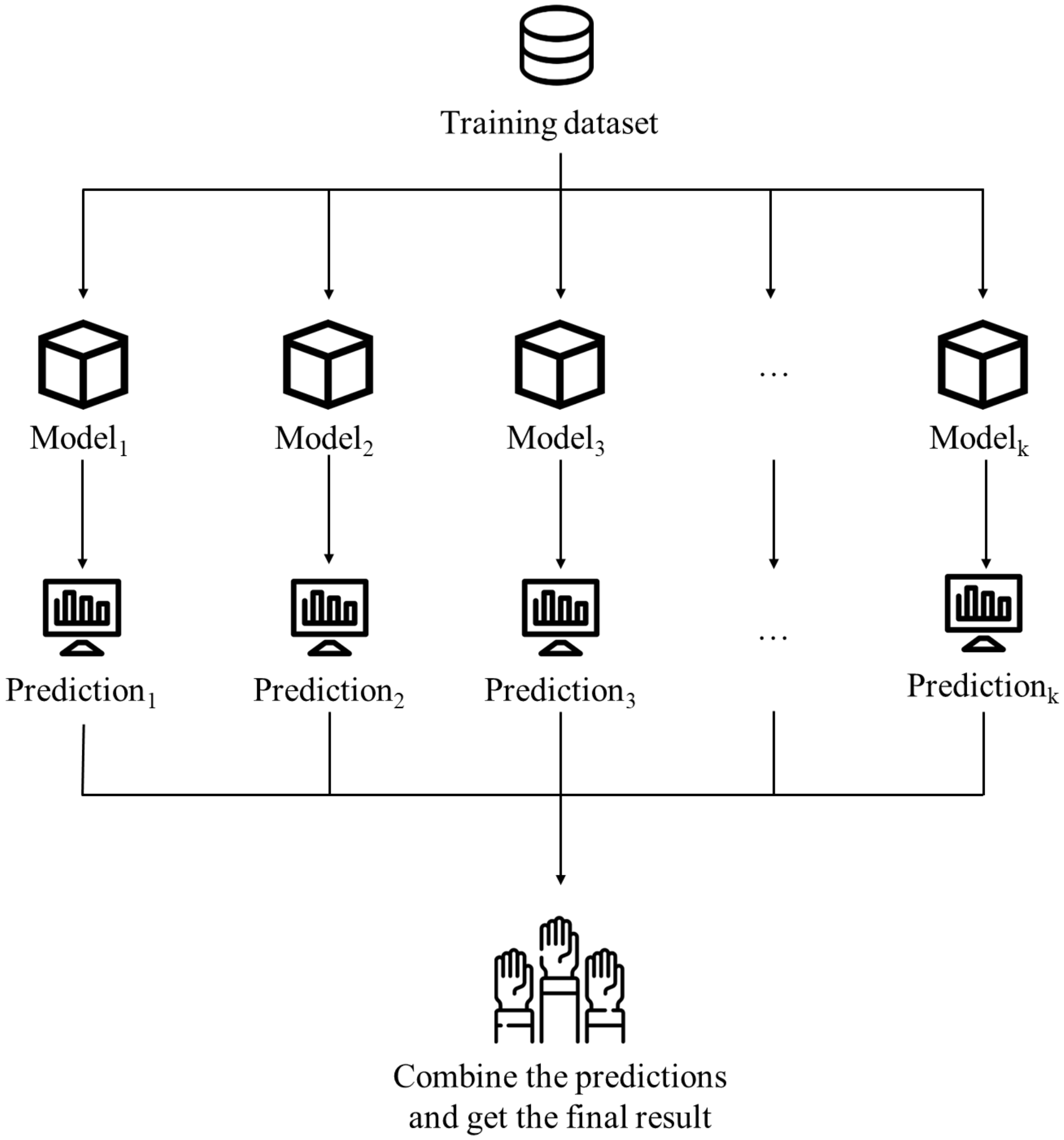

Supplementary Figure 3. Illustration of ensemble learning method.

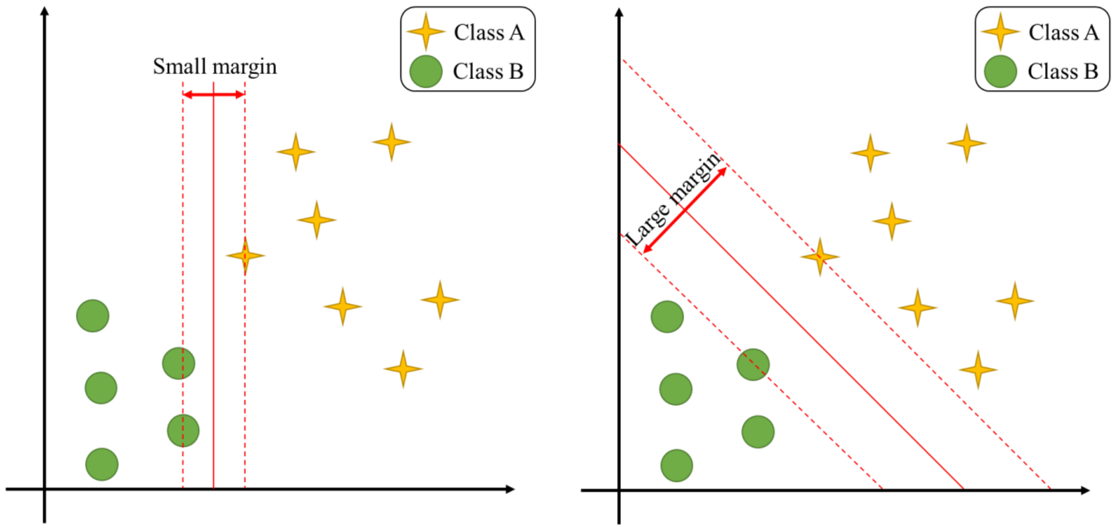

Supplementary Figure 4. Example of possible hyperplanes in SVM.

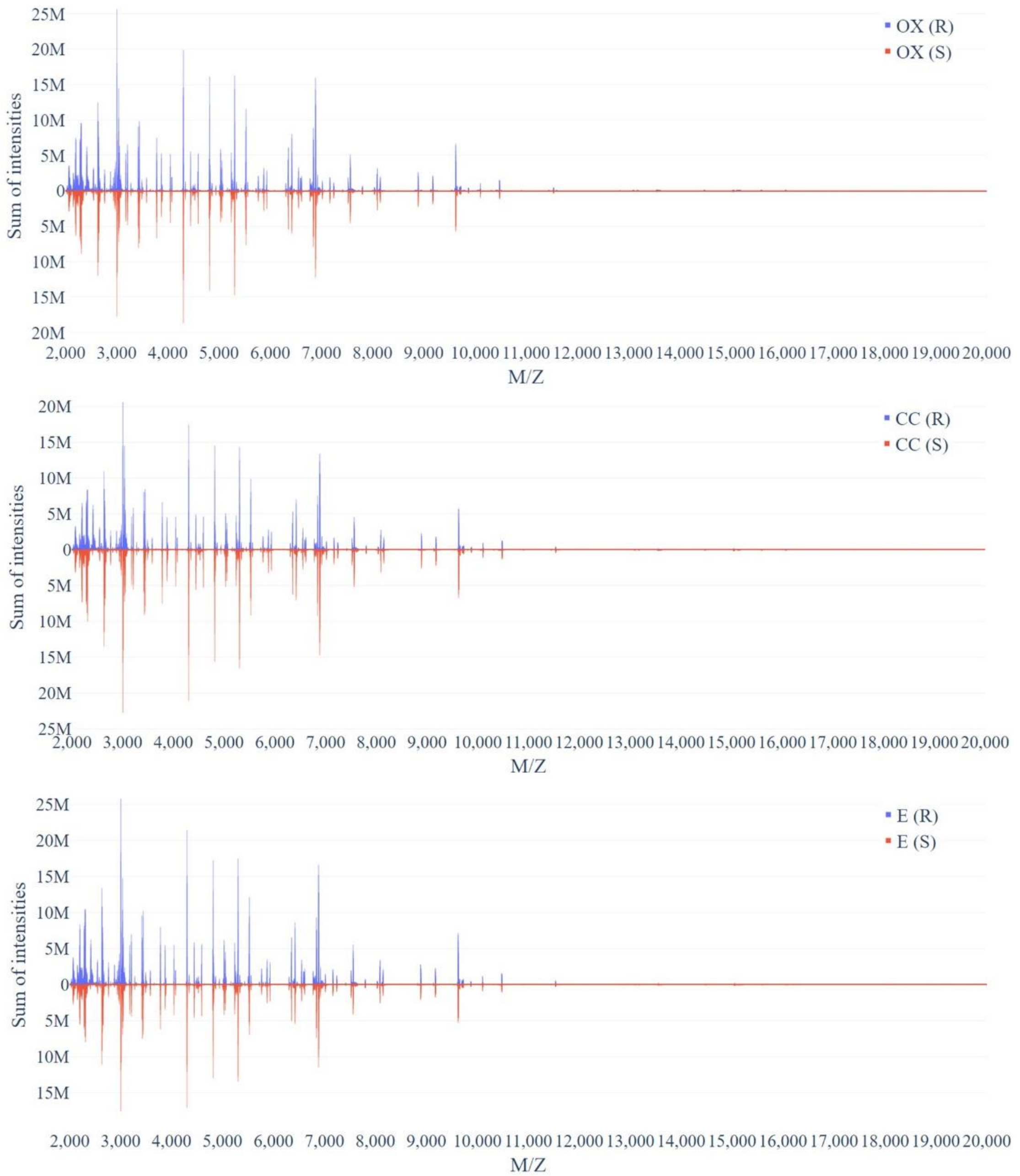

Supplementary Figure 5. Intensity distribution of spectra which were derived from oxacilin- (upper), clindamycin- (middle), and erythromycin-resistant/susceptible (bottom) *Staphylococcus aureus* isolates at each M/Z.

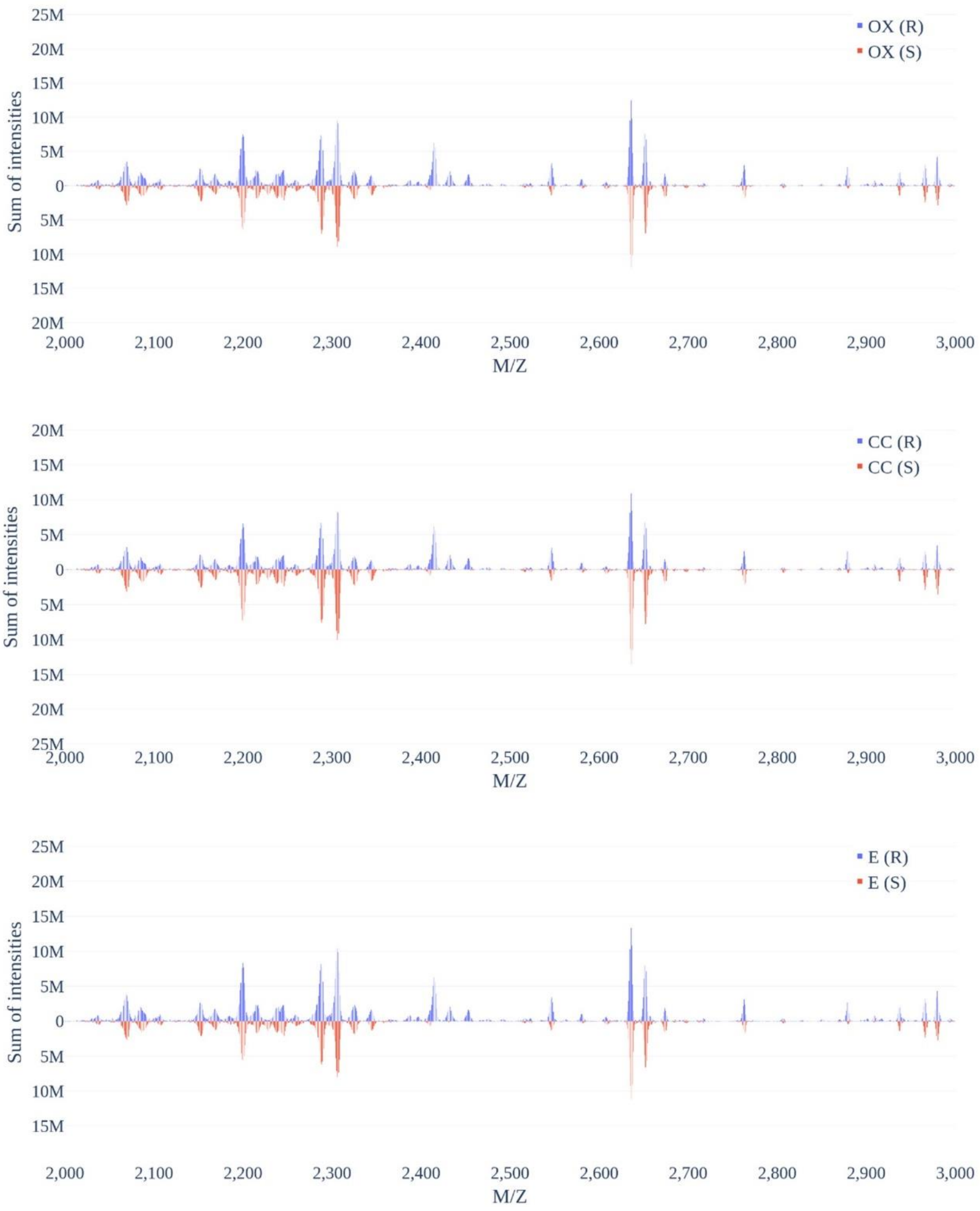

Supplementary Figure 6. Intensity distribution of spectra which were derived from oxacilin- (upper), clindamycin- (middle), and erythromycin-resistant/susceptible (bottom) *Staphylococcus aureus* isolates at M/Z = 2000 to 3000.

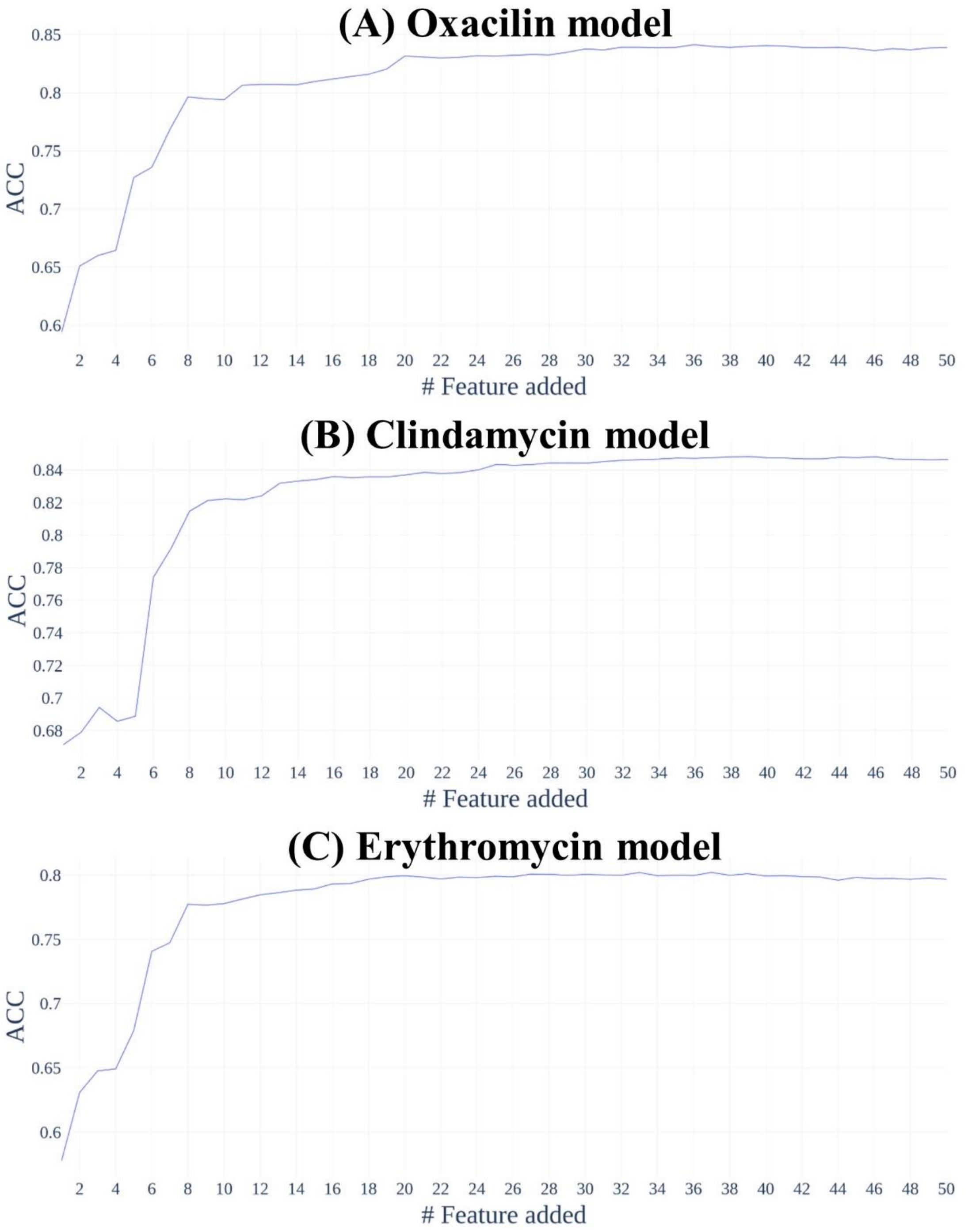

Supplementary Figure 7. Results of forward feature selection for oxacillin (upper), clindamycin (middle), and erythromycin (bottom) models, respectively.

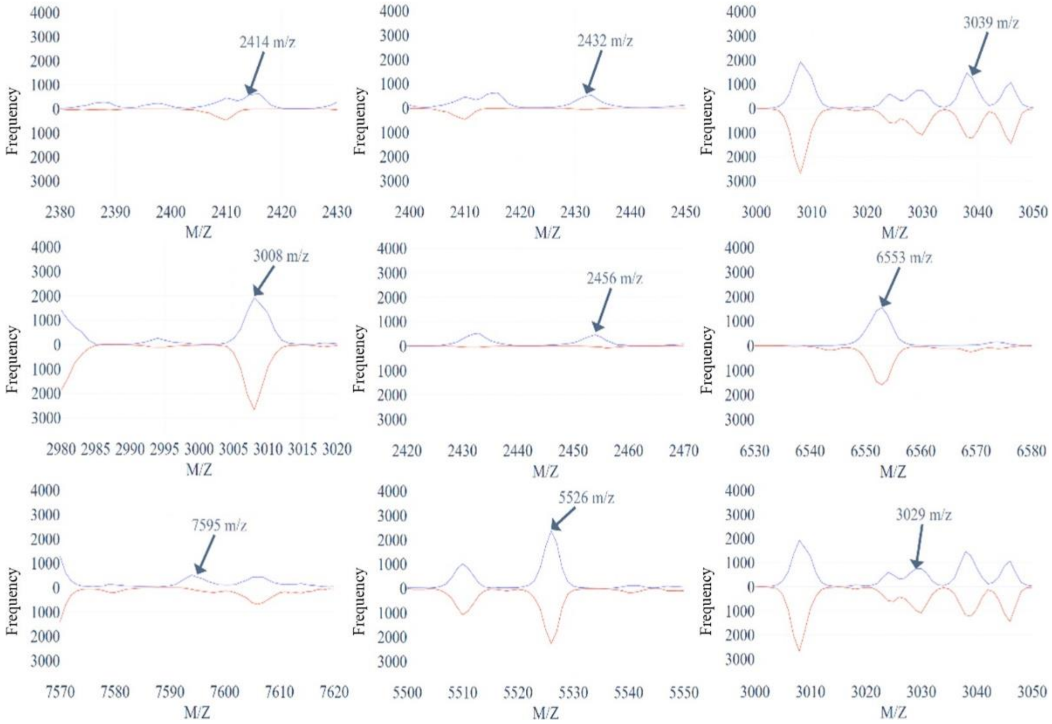

Supplementary Figure 8. The top 9 selected peaks distributions of the M/Z values without peaks alignment for clindamycin -resistant (red)/susceptible (blue) data.

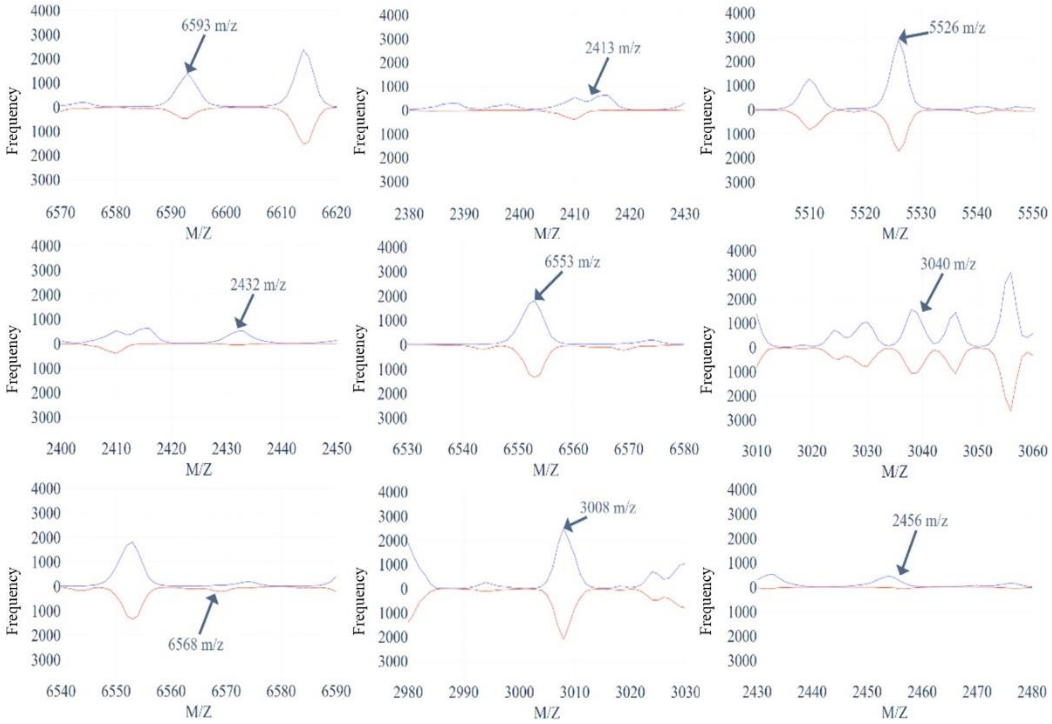

Supplementary Figure 9. The top 9 selected peaks distributions of the M/Z values without peaks alignment for erythromycin-resistant (red)/susceptible (blue) data.

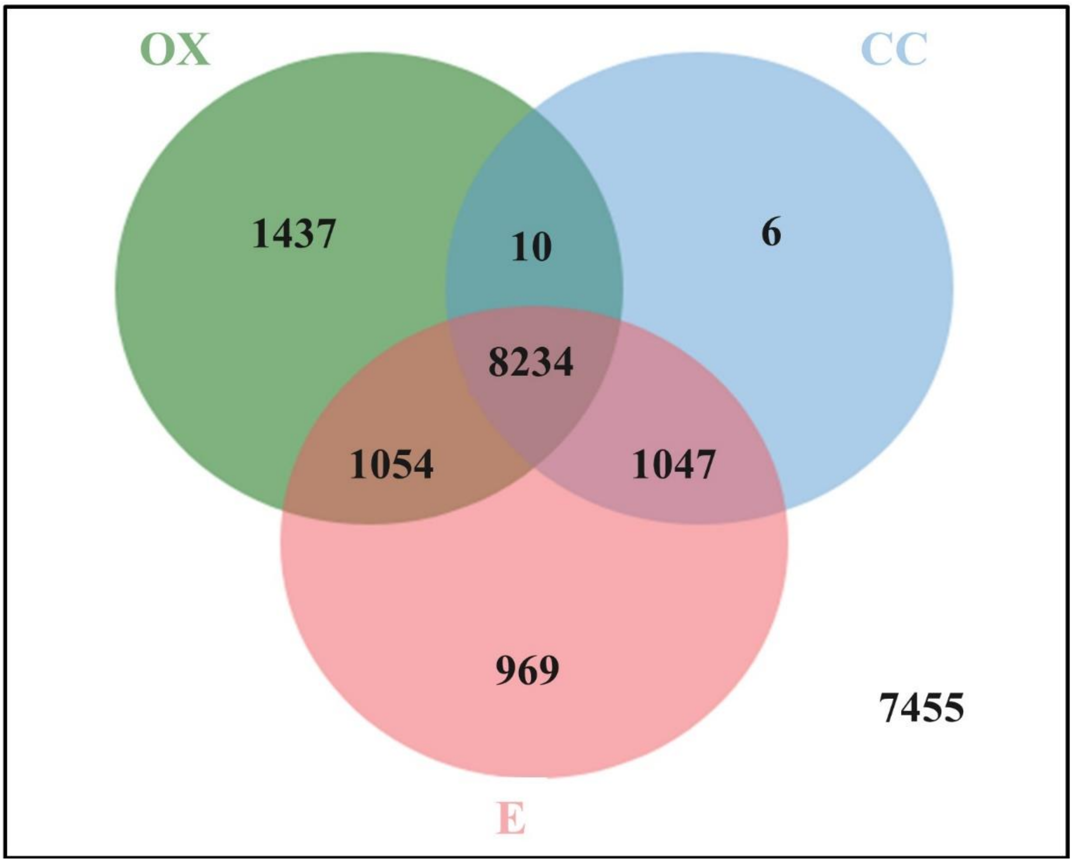

Supplementary Figure 10. Venn diagram for three antibiotics resistant/susceptible data in training set.

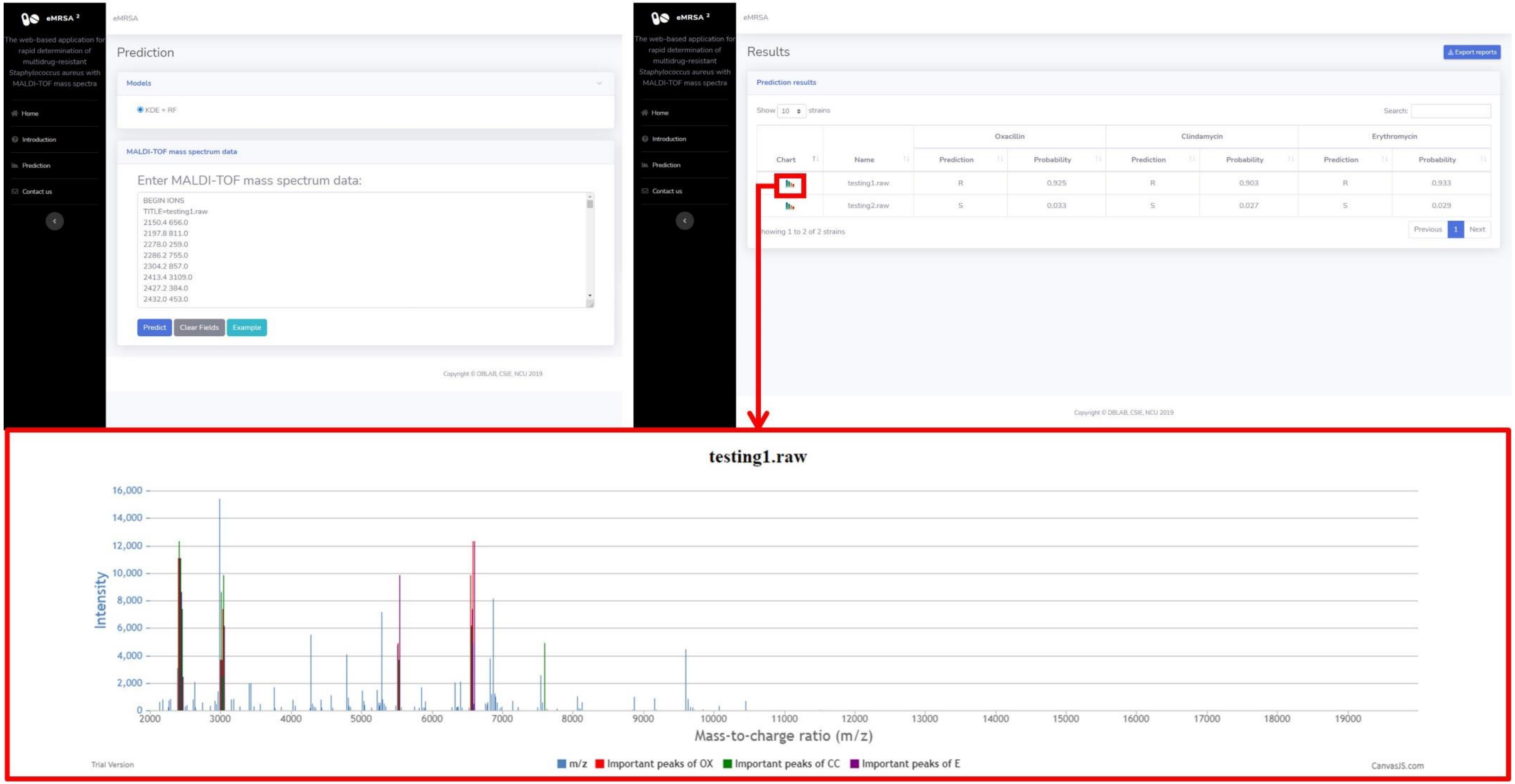

Supplementary Figure 11. Prediction page (upper left), predicted results (upper right), and demonstration of the sumitted MS.

Supplementary Table 1. Demographics statistics in training and independent testing sets.

	**Training set**	**Independent testing set**
Age (mean ± standard deviation)	54.47 ± 24.12	55.15 ± 22.29
Gender		
Female	7984 (39.50%)	2106 (42.08%)
Male	12177 (60.25%)	2896 (57.86%)
Unknown	51 (0.25%)	3 (0.06%)
Specimen types		
Blood	2371 (11.73%)	1091 (21.80%)
Fluid	725 (3.59%)	211 (4.22%)
Respiratory tract	5090 (25.18%)	422 (8.43%)
Urinary tract	103 (0.51%)	15 (0.30%)
Wound	10264 (50.78%)	3222 (64.38)
Others	1659 (8.21%)	44 (0.88%)

Supplementary Table 2. Feature (Peak) list used for different models. There were 1238, 1243, and 1065 peaks were retrieved by KDE for developing oxacilin (OX), clindamycin (CC), and erythromycin (E) models, respectively.

OX	CC	E
2003	2003	2003
2037	2037	2037
2069	2069	2069
2087	2087	2087
2107	2107	2107
2126	2126	2126
2153	2153	2153
2169	2169	2169
2186	2187	2187
2200	2200	2200
2216	2216	2216
2244	2244	2243
2260	2260	2260
2287	2287	2287
2306	2306	2306
2325	2325	2325
2345	2345	2345
2367	2367	2367
2387	2387	2388
2397	2397	2397
2409	2409	2409
2414	2414	2413
2432	2432	2432
2456	2456	2456
2477	2477	2477
2492	2492	2492
2502	2501	2501
2521	2521	2520
2547	2547	2547
2563	2563	2563
2581	2581	2581
2608	2608	2609
2619	2619	2636
2636	2636	2652
2652	2652	2674
2674	2674	2697
2685	2685	2717
2697	2697	2734
2717	2717	2763
2734	2734	2807
2763	2763	2826
2781	2781	2851
2807	2807	2871
2827	2826	2879
2851	2851	2910
2870	2870	2938
2879	2879	2966
2901	2901	2980
2910	2910	2995
2922	2922	3008
2938	2938	3029
2954	2966	3040
2966	2980	3056
2980	2995	3068
2994	3008	3097
3008	3029	3112
3029	3039	3136
3039	3045	3153
3056	3056	3178
3068	3068	3196
3076	3076	3212
3084	3084	3243
3098	3098	3262
3108	3107	3277
3113	3113	3297
3136	3136	3307
3153	3153	3321
3178	3178	3337
3196	3196	3370
3212	3212	3410
3243	3243	3423
3262	3262	3445
3277	3277	3464
3297	3297	3495
3308	3307	3511
3321	3321	3536
3337	3337	3552
3351	3350	3586
3357	3357	3603
3370	3370	3607
3389	3409	3624
3409	3423	3639
3423	3445	3662
3445	3464	3677
3464	3495	3697
3495	3510	3711
3510	3536	3727
3535	3552	3738
3551	3563	3765
3557	3586	3785
3562	3603	3805
3586	3607	3817
3603	3623	3834
3607	3639	3848
3624	3662	3876
3640	3677	3892
3662	3691	3914
3677	3699	3931
3692	3711	3953
3699	3727	3965
3711	3737	3974
3727	3742	3984
3738	3765	4021
3765	3785	4047
3785	3805	4060
3805	3817	4076
3817	3834	4096

3834	3847	4103
3847	3876	4121
3876	3892	4131
3892	3914	4142
3914	3931	4162
3931	3954	4176
3952	3962	4229
3962	3967	4246
3966	3974	4275
3974	3984	4293
3984	4002	4307
4003	4022	4330
4021	4047	4344
4047	4061	4367
4061	4076	4387
4076	4096	4415
4095	4103	4426
4104	4117	4430
4121	4121	4447
4133	4132	4486
4142	4142	4498
4153	4153	4514
4163	4162	4525
4176	4176	4540
4208	4202	4567
4216	4208	4591
4228	4216	4626
4246	4228	4642
4266	4246	4664
4275	4267	4690
4293	4275	4702
4307	4293	4744
4321	4307	4760
4329	4321	4770
4345	4330	4780
4367	4345	4797
4387	4367	4815
4395	4386	4829
4415	4395	4849
4426	4415	4864
4430	4426	4900

4447	4430	4921
4457	4447	4939
4486	4457	4965
4498	4486	4976
4513	4498	4988
4526	4513	5004
4540	4526	5019
4568	4540	5033
4591	4568	5054
4604	4591	5071
4608	4604	5096
4627	4608	5116
4642	4627	5139
4664	4642	5149
4678	4664	5163
4690	4677	5177
4702	4690	5203
4724	4702	5221
4745	4724	5242
4759	4745	5261
4771	4759	5275
4780	4770	5290
4797	4780	5304
4815	4797	5320
4829	4815	5342
4848	4829	5361
4864	4848	5383
4878	4864	5402
4885	4879	5423
4901	4885	5438
4921	4901	5456
4939	4921	5464
4966	4939	5479
4977	4966	5494
4988	4978	5510
5004	4988	5526
5018	5004	5542
5033	5019	5549
5054	5033	5564
5071	5054	5584
5097	5071	5601

5116	5097	5615
5139	5116	5625
5149	5140	5639
5163	5150	5657
5178	5163	5672
5203	5178	5686
5221	5203	5698
5242	5221	5725
5261	5242	5750
5275	5261	5771
5290	5275	5800
5304	5290	5813
5321	5304	5845
5331	5321	5861
5342	5331	5874
5361	5342	5890
5383	5361	5918
5398	5383	5933
5402	5401	5950
5409	5410	5971
5423	5425	5993
5438	5438	6009
5455	5455	6034
5464	5464	6047
5480	5480	6054
5494	5496	6072
5510	5510	6090
5526	5526	6119
5541	5541	6129
5548	5549	6151
5564	5564	6169
5584	5584	6184
5601	5601	6201
5615	5615	6212
5625	5624	6226
5641	5639	6256
5657	5643	6293
5672	5657	6309
5685	5672	6322
5698	5687	6340
5725	5698	6354

5750	5725	6383
5771	5750	6394
5800	5771	6408
5813	5800	6424
5832	5813	6438
5845	5832	6463
5860	5845	6482
5874	5860	6500
5890	5874	6513
5902	5890	6519
5918	5902	6524
5933	5918	6553
5950	5933	6568
5971	5950	6573
5993	5971	6593
6009	5994	6614
6033	6009	6634
6047	6033	6654
6055	6048	6683
6072	6055	6703
6076	6072	6724
6090	6076	6746
6107	6090	6761
6119	6119	6780
6132	6132	6799
6150	6151	6812
6170	6169	6817
6184	6184	6845
6200	6201	6874
6212	6211	6890
6226	6226	6905
6243	6243	6928
6256	6256	6944
6279	6278	6965
6293	6293	6989
6309	6308	7004
6322	6322	7021
6339	6339	7036
6354	6354	7061
6383	6383	7078
6394	6394	7091

6408	6408	7096
6424	6424	7109
6438	6438	7139
6463	6463	7170
6482	6482	7207
6500	6500	7224
6512	6512	7245
6520	6520	7274
6524	6524	7296
6537	6537	7307
6553	6553	7323
6569	6569	7352
6574	6574	7386
6593	6593	7420
6614	6614	7437
6634	6634	7460
6645	6645	7479
6654	6654	7504
6683	6684	7527
6703	6703	7552
6715	6715	7568
6724	6724	7595
6745	6745	7606
6761	6761	7629
6780	6780	7645
6798	6798	7662
6812	6812	7688
6818	6818	7708
6845	6845	7732
6861	6861	7746
6873	6873	7763
6890	6890	7768
6905	6905	7802
6928	6928	7831
6944	6944	7842
6965	6965	7857
6989	6989	7872
7004	7004	7894
7021	7021	7900
7036	7036	7909
7061	7061	7922

7078	7079	7937
7093	7091	7948
7098	7099	7965
7126	7108	8005
7139	7126	8042
7153	7139	8077
7170	7153	8093
7207	7170	8108
7223	7208	8120
7245	7224	8135
7274	7245	8151
7296	7260	8169
7307	7274	8190
7323	7296	8230
7334	7307	8260
7352	7323	8279
7365	7334	8283
7386	7352	8299
7406	7365	8310
7420	7386	8323
7437	7406	8354
7450	7420	8360
7460	7437	8371
7470	7450	8396
7479	7459	8405
7504	7469	8412
7527	7480	8430
7538	7504	8446
7552	7527	8459
7568	7538	8489
7595	7552	8513
7606	7568	8517
7629	7595	8524
7645	7606	8537
7662	7629	8549
7688	7645	8565
7708	7661	8588
7732	7689	8606
7747	7708	8623
7764	7732	8660
7772	7746	8672

7780	7751	8685
7802	7766	8689
7833	7802	8697
7842	7832	8724
7857	7844	8736
7872	7858	8747
7887	7873	8765
7893	7887	8769
7900	7893	8787
7908	7901	8811
7921	7909	8836
7926	7922	8849
7937	7937	8877
7948	7948	8893
7965	7965	8931
7994	7994	8957
8005	8005	8966
8041	8041	8995
8077	8077	9008
8092	8093	9021
8108	8108	9031
8120	8120	9038
8135	8135	9050
8151	8151	9074
8169	8168	9092
8190	8190	9116
8206	8206	9140
8230	8230	9165
8260	8260	9181
8282	8279	9218
8292	8283	9243
8299	8292	9256
8310	8298	9277
8323	8311	9295
8360	8324	9303
8371	8343	9325
8381	8354	9345
8400	8360	9356
8406	8371	9360
8412	8381	9379
8428	8389	9398

8445	8396	9407
8454	8401	9425
8459	8405	9439
8474	8412	9447
8489	8429	9453
8513	8446	9465
8517	8459	9480
8525	8475	9509
8539	8489	9517
8550	8513	9538
8565	8517	9557
8589	8527	9589
8606	8538	9613
8622	8549	9629
8659	8565	9656
8672	8580	9705
8688	8588	9725
8698	8606	9758
8722	8622	9771
8726	8659	9799
8737	8672	9818
8742	8685	9832
8748	8689	9878
8765	8698	9916
8770	8720	9932
8785	8724	9949
8811	8737	9955
8836	8741	9967
8849	8747	9984
8877	8757	9998
8893	8764	10014
8931	8770	10022
8956	8786	10047
8967	8811	10065
8995	8835	10089
9008	8849	10107
9015	8877	10142
9023	8893	10171
9032	8931	10205
9036	8957	10214
9049	8968	10233

9074	8995	10265
9092	9008	10297
9115	9021	10305
9120	9031	10324
9140	9038	10338
9165	9050	10356
9181	9074	10370
9218	9092	10385
9243	9117	10415
9256	9140	10441
9277	9165	10467
9294	9181	10483
9299	9219	10517
9312	9242	10543
9325	9256	10558
9337	9273	10577
9345	9280	10608
9357	9294	10635
9369	9303	10653
9378	9312	10694
9382	9325	10700
9397	9337	10712
9409	9346	10731
9427	9357	10746
9438	9371	10755
9448	9379	10765
9453	9396	10790
9468	9401	10802
9480	9408	10815
9507	9415	10839
9516	9425	10865
9538	9439	10878
9558	9447	10908
9588	9452	10939
9613	9466	10949
9629	9480	10955
9656	9497	10966
9705	9509	10980
9725	9516	10997
9738	9538	11003
9757	9558	11022

9771	9589	11032
9799	9613	11042
9818	9629	11065
9833	9656	11084
9843	9705	11099
9859	9725	11111
9878	9738	11118
9905	9758	11123
9916	9771	11140
9920	9799	11157
9927	9818	11179
9932	9833	11187
9949	9845	11195
9955	9859	11201
9969	9878	11221
9986	9905	11230
9998	9918	11245
10014	9932	11254
10023	9941	11269
10047	9948	11288
10064	9954	11307
10089	9967	11321
10107	9985	11327
10142	9998	11338
10171	10014	11352
10204	10022	11371
10213	10047	11375
10233	10064	11393
10263	10089	11400
10286	10107	11448
10297	10142	11481
10305	10171	11501
10324	10206	11505
10338	10215	11539
10352	10233	11577
10359	10244	11598
10370	10260	11625
10378	10264	11646
10387	10287	11659
10414	10297	11670
10441	10305	11678

10467	10324	11694
10483	10338	11705
10517	10356	11715
10543	10370	11743
10558	10386	11749
10579	10414	11765
10596	10441	11771
10608	10467	11781
10622	10483	11826
10630	10517	11843
10636	10543	11895
10643	10558	11913
10655	10578	11989
10692	10591	12018
10699	10597	12027
10712	10608	12055
10726	10622	12070
10733	10633	12082
10746	10649	12086
10755	10655	12106
10765	10695	12124
10788	10699	12137
10793	10712	12147
10802	10726	12158
10812	10733	12183
10818	10746	12190
10828	10755	12197
10838	10765	12217
10843	10788	12227
10866	10793	12245
10877	10802	12288
10908	10815	12301
10926	10828	12318
10940	10838	12342
10951	10864	12369
10955	10877	12388
10966	10908	12393
10980	10940	12413
10998	10950	12432
11002	10955	12441
11022	10966	12445

11032	10980	12466
11045	10998	12481
11064	11022	12508
11082	11032	12544
11099	11042	12549
11116	11064	12583
11122	11082	12614
11140	11094	12629
11157	11101	12661
11181	11116	12691
11187	11122	12711
11195	11140	12715
11200	11157	12726
11204	11181	12731
11221	11195	12747
11230	11204	12773
11250	11221	12778
11255	11230	12837
11259	11250	12883
11269	11255	12893
11286	11259	12929
11290	11269	12988
11307	11286	12992
11317	11290	13022
11321	11307	13037
11327	11327	13099
11338	11338	13128
11351	11351	13182
11363	11361	13223
11372	11372	13235
11378	11391	13242
11392	11398	13256
11398	11411	13277
11403	11422	13285
11410	11448	13297
11421	11481	13302
11448	11504	13312
11469	11539	13321
11480	11576	13329
11504	11598	13349
11539	11625	13355

11552	11646	13364
11576	11659	13377
11598	11671	13383
11625	11678	13387
11646	11687	13400
11659	11694	13411
11671	11704	13420
11678	11716	13426
11687	11736	13438
11695	11743	13452
11705	11750	13462
11716	11770	13474
11744	11776	13519
11750	11781	13557
11770	11827	13588
11776	11843	13615
11781	11895	13659
11827	11913	13674
11843	11990	13691
11895	12018	13697
11913	12027	13729
11989	12044	13744
12018	12055	13776
12027	12070	13806
12044	12086	13814
12055	12106	13832
12070	12123	13848
12082	12137	13866
12086	12147	13891
12106	12151	13909
12123	12157	13920
12137	12183	13936
12147	12190	13948
12151	12197	13956
12157	12217	13961
12183	12227	13982
12190	12245	13993
12197	12288	13998
12215	12302	14012
12221	12318	14048
12226	12342	14058

12245	12370	14085
12288	12387	14098
12302	12397	14108
12318	12414	14119
12342	12429	14138
12370	12445	14166
12387	12466	14196
12394	12479	14200
12400	12484	14208
12414	12494	14220
12431	12508	14233
12439	12544	14268
12445	12549	14273
12466	12584	14296
12479	12614	14304
12484	12629	14314
12508	12661	14373
12547	12691	14392
12584	12713	14405
12610	12726	14448
12615	12731	14485
12629	12747	14535
12661	12767	14543
12689	12774	14552
12693	12779	14557
12713	12810	14587
12726	12837	14652
12731	12863	14656
12747	12869	14682
12767	12883	14701
12775	12893	14729
12811	12929	14734
12837	12988	14747
12862	12993	14767
12869	13022	14774
12883	13037	14787
12893	13055	14798
12929	13065	14814
12984	13099	14824
12992	13124	14840
13022	13128	14856

13037	13182	14872
13054	13213	14876
13099	13222	14894
13127	13233	14913
13143	13242	14923
13182	13254	14930
13211	13262	14943
13222	13277	14958
13232	13287	14976
13242	13297	14980
13253	13302	14990
13260	13312	15007
13277	13319	15016
13300	13327	15050
13306	13331	15154
13314	13350	15182
13319	13355	15221
13327	13364	15284
13333	13372	15296
13349	13378	15307
13353	13383	15322
13358	13387	15336
13363	13400	15350
13367	13410	15359
13372	13420	15363
13378	13427	15371
13383	13438	15375
13387	13451	15379
13400	13459	15388
13410	13463	15397
13420	13474	15409
13429	13518	15424
13438	13557	15435
13449	13588	15450
13462	13615	15463
13473	13653	15471
13488	13660	15491
13519	13673	15498
13557	13677	15516
13588	13684	15527
13615	13697	15599

13643	13729	15647
13660	13744	15655
13672	13777	15664
13676	13794	15670
13687	13802	15684
13698	13806	15691
13726	13812	15701
13730	13817	15713
13744	13832	15731
13777	13848	15745
13794	13865	15753
13802	13874	15762
13806	13890	15771
13814	13897	15782
13819	13908	15792
13832	13920	15807
13845	13926	15822
13849	13936	15833
13853	13948	15850
13865	13954	15870
13876	13961	15897
13889	13985	15912
13909	13992	15919
13920	13998	15924
13926	14012	15945
13936	14032	15960
13948	14046	15977
13961	14054	16008
13985	14085	16075
13992	14097	16122
13998	14108	16141
14012	14119	16149
14032	14138	16192
14046	14166	16196
14054	14196	16204
14085	14200	16211
14097	14209	16261
14108	14220	16266
14119	14233	16319
14138	14270	16332
14166	14296	16348

14196	14304	16372
14200	14314	16381
14209	14373	16398
14220	14392	16403
14233	14405	16415
14268	14421	16450
14273	14449	16455
14297	14485	16479
14304	14535	16512
14314	14542	16544
14373	14551	16562
14392	14556	16569
14405	14587	16575
14421	14653	16584
14448	14678	16595
14485	14683	16600
14533	14701	16604
14542	14729	16615
14551	14734	16622
14556	14747	16644
14563	14767	16653
14584	14774	16659
14588	14785	16671
14613	14789	16680
14653	14799	16688
14682	14808	16710
14701	14813	16719
14729	14819	16733
14734	14825	16773
14747	14840	16781
14767	14848	16786
14774	14856	16801
14785	14870	16815
14789	14876	16839
14799	14881	16848
14814	14897	16853
14823	14905	16866
14827	14909	16879
14843	14923	16887
14854	14930	16896
14858	14942	16906

14877	14959	16928
14897	14975	16949
14909	14979	16963
14915	14990	16974
14925	15007	16985
14931	15015	16990
14944	15050	16996
14959	15131	17014
14967	15154	17019
14975	15182	17031
14980	15186	17035
14990	15217	17041
15007	15221	17059
15011	15253	17070
15015	15264	17077
15050	15270	17081
15095	15286	17093
15155	15296	17105
15182	15308	17133
15220	15321	17148
15253	15337	17158
15268	15347	17181
15285	15353	17189
15296	15362	17200
15304	15370	17204
15308	15378	17209
15323	15388	17213
15337	15398	17223
15346	15410	17243
15359	15424	17260
15363	15435	17270
15371	15443	17285
15375	15450	17297
15379	15463	17301
15385	15470	17313
15391	15490	17318
15407	15499	17340
15411	15516	17356
15424	15527	17364
15436	15532	17373
15450	15599	17384

15464	15646	17396
15469	15654	17407
15477	15665	17413
15498	15672	17419
15516	15684	17433
15525	15692	17446
15530	15701	17460
15599	15713	17472
15646	15721	17492
15653	15731	17500
15670	15743	17510
15685	15753	17523
15692	15762	17528
15702	15770	17552
15712	15782	17557
15722	15793	17563
15731	15806	17574
15745	15824	17587
15753	15832	17598
15762	15849	17607
15770	15870	17612
15782	15886	17620
15795	15890	17655
15806	15897	17660
15821	15912	17705
15826	15920	17715
15833	15924	17720
15848	15936	17731
15855	15944	17740
15870	15951	17745
15884	15959	17751
15890	15971	17765
15897	15977	17774
15911	16003	17778
15920	16009	17791
15933	16015	17804
15938	16075	17815
15944	16119	17832
15954	16127	17842
15962	16142	17856
15979	16152	17877

16003	16165	17887
16009	16174	17902
16014	16183	17911
16042	16191	17928
16076	16196	17937
16111	16204	17951
16118	16208	17962
16125	16235	17971
16142	16242	17978
16147	16260	17996
16153	16267	18003
16164	16300	18016
16198	16309	18021
16208	16318	18034
16215	16332	18040
16260	16349	18058
16267	16372	18062
16308	16382	18066
16315	16397	18074
16319	16406	18081
16332	16415	18098
16349	16428	18104
16360	16451	18119
16368	16458	18133
16372	16474	18139
16381	16481	18153
16397	16490	18158
16406	16499	18163
16415	16512	18173
16428	16538	18183
16452	16546	18195
16458	16561	18211
16475	16574	18220
16482	16584	18232
16500	16595	18239
16513	16604	18254
16541	16615	18270
16547	16623	18277
16562	16638	18295
16574	16644	18307
16584	16652	18326

16596	16659	18339
16604	16668	18348
16615	16672	18366
16624	16679	18371
16630	16685	18375
16647	16689	18390
16653	16698	18413
16659	16709	18424
16665	16719	18441
16669	16733	18446
16679	16780	18455
16688	16786	18463
16698	16802	18468
16710	16815	18484
16719	16840	18492
16733	16848	18503
16773	16853	18516
16781	16866	18529
16787	16879	18535
16802	16887	18547
16812	16896	18555
16817	16906	18559
16840	16930	18570
16848	16938	18584
16853	16949	18602
16866	16962	18615
16879	16971	18624
16887	16975	18628
16896	16986	18650
16906	16990	18659
16928	16997	18663
16938	17013	18675
16949	17020	18682
16963	17033	18694
16971	17042	18706
16975	17059	18721
16988	17069	18738
16992	17077	18745
16996	17081	18755
17013	17087	18760
17019	17093	18773

17033	17104	18784
17042	17113	18796
17060	17133	18800
17069	17148	18815
17077	17158	18820
17081	17182	18827
17093	17191	18836
17105	17199	18845
17113	17205	18851
17126	17209	18861
17132	17213	18876
17148	17223	18883
17159	17243	18892
17182	17257	18904
17191	17265	18914
17199	17271	18922
17205	17287	18931
17214	17297	18935
17223	17301	18946
17243	17313	18974
17256	17317	18998
17263	17329	19010
17270	17340	19014
17287	17355	19033
17297	17365	19042
17301	17373	19051
17317	17384	19055
17322	17396	19062
17330	17407	19071
17340	17414	19078
17355	17423	19092
17366	17435	19097
17373	17446	19105
17384	17458	19119
17396	17463	19130
17408	17472	19139
17417	17492	19153
17434	17498	19160
17448	17507	19174
17457	17511	19189
17464	17523	19201

17473	17528	19214
17491	17536	19220
17499	17546	19230
17510	17553	19240
17523	17557	19256
17528	17563	19264
17536	17574	19276
17543	17586	19280
17551	17598	19285
17556	17604	19291
17563	17611	19310
17575	17620	19319
17585	17651	19336
17598	17660	19340
17613	17705	19347
17622	17719	19357
17659	17731	19362
17705	17739	19375
17719	17748	19390
17731	17752	19395
17738	17765	19407
17744	17773	19413
17750	17778	19425
17765	17790	19438
17773	17798	19450
17778	17802	19462
17790	17815	19476
17800	17831	19486
17816	17842	19492
17831	17855	19501
17842	17865	19513
17855	17877	19526
17865	17886	19540
17879	17897	19558
17886	17902	19573
17900	17912	19582
17913	17928	19589
17928	17937	19595
17938	17950	19605
17948	17962	19619
17953	17971	19634

17962	17977	19653
17971	17990	19676
17977	17997	19693
17991	18004	19698
18004	18015	19712
18015	18020	19717
18021	18025	19736
18034	18034	19749
18041	18041	19770
18056	18056	19774
18065	18066	19788
18075	18076	19798
18081	18080	19805
18100	18098	19857
18107	18107	19998
18118	18118	
18134	18124	
18140	18134	
18154	18140	
18158	18154	
18163	18163	
18174	18172	
18195	18182	
18211	18195	
18220	18211	
18227	18220	
18232	18227	
18241	18232	
18245	18239	
18256	18245	
18269	18254	
18278	18258	
18295	18267	
18306	18271	
18310	18278	
18326	18295	
18339	18307	
18347	18326	
18355	18339	
18366	18347	
18372	18355	

18379	18366	
18390	18371	
18408	18375	
18418	18390	
18422	18408	
18426	18414	
18433	18425	
18442	18432	
18462	18442	
18466	18454	
18470	18465	
18480	18485	
18485	18492	
18492	18503	
18503	18513	
18513	18518	
18518	18529	
18529	18535	
18536	18539	
18541	18547	
18548	18559	
18559	18570	
18570	18585	
18581	18602	
18585	18615	
18602	18626	
18615	18637	
18626	18643	
18637	18651	
18645	18663	
18650	18675	
18663	18688	
18675	18695	
18681	18706	
18688	18721	
18695	18739	
18707	18747	
18713	18758	
18721	18774	
18739	18785	
18751	18794	

18759	18801	
18774	18814	
18785	18820	
18795	18827	
18800	18836	
18814	18845	
18820	18852	
18827	18861	
18836	18876	
18845	18883	
18851	18892	
18861	18904	
18876	18915	
18883	18922	
18890	18931	
18894	18935	
18904	18946	
18914	18953	
18922	18965	
18936	18975	
18946	18985	
18965	18989	
18974	18995	
18983	18999	
18989	19014	
18998	19030	
19013	19034	
19027	19044	
19034	19051	
19043	19055	
19052	19062	
19056	19071	
19066	19078	
19070	19092	
19077	19097	
19092	19106	
19097	19114	
19105	19119	
19117	19129	
19129	19138	
19142	19144	

19152	19153	
19156	19160	
19164	19164	
19172	19170	
19183	19175	
19197	19186	
19203	19197	
19214	19203	
19230	19214	
19240	19220	
19257	19228	
19264	19240	
19280	19257	
19286	19264	
19291	19279	
19318	19286	
19324	19292	
19335	19310	
19340	19319	
19348	19335	
19362	19339	
19375	19347	
19388	19357	
19396	19362	
19408	19375	
19412	19387	
19425	19396	
19439	19413	
19444	19425	
19452	19438	
19461	19445	
19476	19452	
19486	19461	
19493	19476	
19501	19486	
19513	19493	
19526	19501	
19540	19513	
19553	19525	
19558	19540	
19562	19553	

19574	19558	
19583	19562	
19594	19573	
19606	19583	
19613	19589	
19625	19595	
19634	19604	
19647	19608	
19654	19612	
19676	19619	
19692	19625	
19698	19635	
19703	19647	
19714	19656	
19723	19676	
19735	19692	
19748	19703	
19766	19715	
19770	19723	
19788	19735	
19798	19748	
19805	19766	
19857	19770	
19998	19777	
	19788	
	19798	
	19805	
	19857	
	19998	

Supplementary Table 3. Results of 10-fold cross validation with the optimal parameters based on the grid search for (A) oxacillin, (B) clindamycin, and (C) erythromycin models for different bandwidths (bw_method). Note that the bold ones are chosen as the optimal one with lower fluctuations.

(A) Oxacillin model

bw_method	#Features	Model	SN	SP	ACC	AUC
0.0006	786	DT	0.8168±0.0351	0.8368±0.0394	0.8261±0.0102	0.9013±0.0079
		RF	0.8722±0.0214	0.8584±0.0209	0.8657±0.0071	0.9332±0.0066
		SVM	0.8551±0.0193	0.8374±0.0347	0.8468±0.0088	0.9132±0.0072
0.0008	901	DT	0.8203±0.0283	0.8216±0.0345	0.8209±0.0094	0.8960±0.0079
		RF	**0.8606±0.0148**	**0.8682±0.0166**	**0.8642±0.0071**	**0.9330±0.0064**
		SVM	0.8478±0.0331	0.8382±0.0307	0.8433±0.0100	0.9119±0.0067
0.0010	1068	DT	0.8060±0.0315	0.8338±0.0325	0.8190±0.0116	0.8950±0.0083
		RF	0.8740±0.0242	0.8533±0.0274	0.8643±0.0078	0.9324±0.0068
		SVM	0.8416±0.0274	0.8419±0.0180	0.8417±0.0110	0.9097±0.0076
0.0012	1238	DT	0.8042±0.0161	0.8218±0.0168	0.8124±0.0095	0.8905±0.0072
		RF	0.8645±0.0137	0.8466±0.0188	0.8561±0.0078	0.9282±0.0068
		SVM	0.8430±0.0286	0.8333±0.0321	0.8385±0.0092	0.9067±0.0082
0.0014	1500	DT	0.7844±0.0571	0.8086±0.0472	0.7958±0.0106	0.8789±0.0089
		RF	0.8473±0.0289	0.8495±0.0196	0.8484±0.0104	0.9245±0.0069
		SVM	0.8377±0.0222	0.8216±0.0322	0.8301±0.0098	0.8995±0.0077

(B) Clindamycin model						
**bw_method**	**#Features**	**Model**	**SN**	**SP**	**ACC**	**AUC**
0.0006	786	DT	0.7808±0.0247	0.8626±0.0236	0.8250±0.0092	0.8869±0.0067
		RF	0.8119±0.0180	0.9013±0.0218	0.8602±0.0106	0.9175±0.0076
		SVM	0.8010±0.0254	0.8738±0.0215	0.8403±0.0073	0.9017±0.0061
0.0008	901	DT	0.7663±0.0310	0.8749±0.0334	0.8249±0.0106	0.8862±0.0077
		RF	**0.8140±0.0196**	**0.9000±0.0128**	**0.8605±0.0093**	**0.9174±0.0076**
		SVM	0.8171±0.0234	0.8557±0.0195	0.8380±0.0091	0.8996±0.0062
0.0010	1068	DT	0.7821±0.0219	0.8568±0.0242	0.8224±0.0112	0.8847±0.0095
		RF	0.8062±0.0322	0.9037±0.0235	0.8588±0.0109	0.9171±0.0079
		SVM	0.8043±0.0237	0.8681±0.0191	0.8388±0.0074	0.8977±0.0072
0.0012	1238	DT	0.7720±0.0291	0.8664±0.0255	0.8230±0.0118	0.8859±0.0107
		RF	0.8067±0.0220	0.9069±0.0212	0.8608±0.0097	0.9173±0.0085
		SVM	0.8168±0.0211	0.8533±0.0150	0.8365±0.0091	0.8961±0.0081
0.0014	1500	DT	0.7684±0.0256	0.8686±0.0295	0.8225±0.0088	0.8835±0.0071
		RF	0.8055±0.0282	0.9000±0.0118	0.8566±0.0099	0.9158±0.0090
		SVM	0.7985±0.0262	0.8665±0.0187	0.8352±0.0081	0.8936±0.0075

(C) Erythromycin model						
**bw_method**	**#Features**	**Model**	**SN**	**SP**	**ACC**	**AUC**
0.0006	786	DT	0.7322±0.0221	0.8515±0.0231	0.7848±0.0060	0.8612±0.0065
		RF	0.7657±0.0258	0.8754±0.0260	0.8140±0.0084	0.8923±0.0040
		SVM	0.7548±0.0310	0.8467±0.0337	0.7953±0.0096	0.8731±0.0063
0.0008	901	DT	0.7323±0.0290	0.8454±0.0151	0.7822±0.0121	0.8578±0.0088
		RF	0.7520±0.0247	0.8852±0.0217	0.8107±0.0079	0.8933±0.0042
		SVM	0.7560±0.0285	0.8454±0.0307	0.7954±0.0097	0.8721±0.0068
0.0010	1068	DT	0.7305±0.0237	0.8524±0.0235	0.7842±0.0073	0.8593±0.0055
		RF	**0.7614±0.0218**	**0.8786±0.0220**	**0.8131±0.0066**	**0.8929±0.0036**
		SVM	0.7570±0.0182	0.8412±0.0272	0.7941±0.0060	0.8711±0.0062
0.0012	1238	DT	0.7395±0.0309	0.8436±0.0211	0.7854±0.0107	0.8607±0.0094
		RF	0.7581±0.0146	0.8798±0.0145	0.8117±0.0071	0.8929±0.0042
		SVM	0.7404±0.0281	0.8569±0.0233	0.7917±0.0091	0.8708±0.0063
0.0014	1500	DT	0.7275±0.0304	0.8326±0.0209	0.7738±0.0127	0.8524±0.0074
		RF	0.7561±0.0233	0.8782±0.0250	0.8099±0.0076	0.8905±0.0053
		SVM	0.7415±0.0292	0.8483±0.0347	0.7886±0.0082	0.8655±0.0060

Supplementary Table 4. The selected features for oxacilin, clinidamycin, and erythromycin models.

	M/Z				M/Z		
**Rank**	**OX**	**CC**	**E**	**Rank**	**OX**	**CC**	**E**
**1**	6593	2414	6593	**21**	3056	6569	6424
**2**	2414	2432	2413	**22**	11539	3056	3056
**3**	6553	3039	5526	**23**	2547	6614	6524
**4**	2432	3008	2432	**24**	2306	15154	6890
**5**	3039	2456	6553	**25**	2287	5033	5290
**6**	6569	6553	3040	**26**	6424	2069	13128
**7**	5526	7595	6568	**27**	13127	3277	4514
**8**	3008	5526	3008	**28**	6905	2397	2287
**9**	2456	3029	2456	**29**	6890	6424	7527
**10**	3765	3076	7595	**30**	4526	6890	6928
**11**	2636	2879	3029	**31**	3297	7527	3445
**12**	3029	2980	3068	**32**	6928	6438	9629
**13**	7595	2636	2980	**33**	5290	2966	5004
**14**	2980	6593	6614	**34**	3445	9629	2652
**15**	6614	2306	6438	**35**	2200	7021	2200
**16**	2879	2910	5033	**36**	3277	7568	7021
**17**	3076	3045	6905	**37**		3765	2069
**18**	5033	2287	2636	**38**		3445	
**19**	6438	2200	2547				
**20**	9629	2547	2306				

Supplementary Table 5. Results of Chi-square tests and t-tests for selected features on oxacilin model.

	Number (Proportion) of spectra			Mean (Standard devation) of intensity		
M/Z	R	S	p-value	R	S	p-value
**6593**	6364 (0.593)	2881 (0.304)	<0.001*	0.061 (0.068)	0.028 (0.055)	<0.001*
**2414**	3174 (0.296)	267 (0.028)	0	0.138 (0.278)	0.004 (0.031)	0
**6553**	8637 (0.805)	6857 (0.724)	<0.001*	0.123 (0.105)	0.116 (0.096)	<0.001*
**2432**	2932 (0.273)	277 (0.029)	0	0.062 (0.146)	0.004 (0.032)	<0.001*
**3039**	8395 (0.782)	6732 (0.710)	<0.001*	0.323 (0.336)	0.240 (0.296)	<0.001*
**6569**	199 (0.019)	1409 (0.149)	<0.001*	0.002 (0.015)	0.018 (0.049)	<0.001*
**5526**	10027 (0.934)	7044 (0.743)	<0.001*	0.341 (0.230)	0.269 (0.233)	<0.001*
**3008**	9321 (0.868)	8032 (0.848)	<0.001*	0.462 (0.396)	0.394 (0.371)	<0.001*
**2456**	2499 (0.233)	300 (0.032)	0	0.044 (0.112)	0.005 (0.031)	<0.001*
**3765**	266 (0.025)	1303 (0.137)	<0.001*	0.002 (0.020)	0.011 (0.037)	<0.001*
**2636**	7510 (0.700)	7013 (0.740)	<0.001*	0.254 (0.292)	0.320 (0.322)	<0.001*
**3029**	5759 (0.536)	4798 (0.506)	<0.001*	0.117 (0.164)	0.093 (0.136)	<0.001*
**7595**	3079 (0.287)	1160 (0.122)	<0.001*	0.020 (0.041)	0.008 (0.029)	<0.001*
**2980**	6931 (0.646)	5634 (0.594)	<0.001*	0.093 (0.102)	0.076 (0.088)	<0.001*
**6614**	9583 (0.893)	7073 (0.746)	<0.001*	0.073 (0.045)	0.066 (0.053)	<0.001*
**2879**	2532 (0.236)	807 (0.085)	<0.001*	0.047 (0.128)	0.007 (0.030)	<0.001*
**3076**	2548 (0.237)	1558 (0.164)	<0.001*	0.036 (0.086)	0.023 (0.071)	<0.001*
**5033**	9776 (0.911)	7686 (0.811)	<0.001*	0.171 (0.116)	0.159 (0.126)	<0.001*
**6438**	218 (0.020)	1607 (0.170)	<0.001*	0.003 (0.026)	0.049 (0.129)	<0.001*
**9629**	10357 (0.965)	9266 (0.978)	<0.001*	0.335 (0.200)	0.365 (0.203)	<0.001*
**3056**	10062 (0.937)	8885 (0.938)	<0.001*	0.185 (0.139)	0.177 (0.133)	<0.001*
**11539**	9537 (0.888)	7066 (0.746)	<0.001*	0.032 (0.023)	0.029 (0.026)	<0.001*
**2547**	4921 (0.458)	3060 (0.323)	<0.001*	0.073 (0.120)	0.041 (0.081)	<0.001*
**2306**	7808 (0.727)	6811 (0.719)	<0.001*	0.301 (0.331)	0.325 (0.335)	<0.001*
**2287**	8106 (0.755)	7107 (0.750)	<0.001*	0.282 (0.305)	0.307 (0.315)	<0.001*
**6424**	10635 (0.991)	9165 (0.967)	<0.001*	0.285 (0.161)	0.272 (0.172)	<0.001*

**13127**	106 (0.010)	743 (0.078)	<0.001*	0.000 (0.003)	0.002 (0.007)	<0.001*
**6905**	717 (0.067)	1935 (0.204)	<0.001*	0.010 (0.058)	0.097 (0.246)	<0.001*
**6890**	10586 (0.986)	8985 (0.948)	<0.001*	0.574 (0.322)	0.545 (0.342)	<0.001*
**4526**	3324 (0.310)	3398 (0.359)	<0.001*	0.028 (0.057)	0.045 (0.091)	<0.001*
**3297**	888 (0.083)	111 (0.012)	<0.001*	0.007 (0.026)	0.001 (0.012)	<0.001*
**6928**	9032 (0.841)	6979 (0.736)	<0.001*	0.103 (0.085)	0.097 (0.090)	<0.001*
**5290**	6044 (0.563)	5674 (0.599)	<0.001*	0.056 (0.066)	0.091 (0.126)	<0.001*
**3445**	10280 (0.958)	9111 (0.961)	<0.001*	0.238 (0.169)	0.253 (0.171)	<0.001*
**2200**	6591 (0.614)	5130 (0.541)	<0.001*	0.244 (0.313)	0.232 (0.315)	<0.001*
**3277**	3186 (0.297)	2864 (0.302)	<0.001*	0.029 (0.053)	0.027 (0.048)	<0.001*

Supplementary Table 6. Results of Chi-square tests and t-tests for selected features on clinidamycin model.

	Number (Proportion) of spectra			Mean (Standard devation) of intensity		
M/Z	R	S	p-value	R	S	p-value
**2414**	3149 (0.339)	292 (0.027)	0	0.159 (0.294)	0.004 (0.032)	0
**2432**	2898 (0.312)	311 (0.028)	0	0.072 (0.156)	0.004 (0.031)	0
**3039**	5385 (0.579)	5038 (0.462)	<0.001*	0.293 (0.370)	0.164 (0.280)	<0.001*
**3008**	7917 (0.852)	9436 (0.864)	<0.001*	0.435 (0.397)	0.427 (0.377)	0.127
**2456**	2443 (0.263)	356 (0.033)	0	0.050 (0.121)	0.005 (0.032)	<0.001*
**6553**	7668 (0.825)	7826 (0.717)	0.204	0.129 (0.107)	0.112 (0.095)	<0.001*
**7595**	2972 (0.320)	1267 (0.116)	<0.001*	0.023 (0.043)	0.008 (0.028)	<0.001*
**5526**	8633 (0.929)	8438 (0.773)	0.136	0.333 (0.232)	0.285 (0.234)	<0.001*
**3029**	4628 (0.498)	5929 (0.543)	<0.001*	0.115 (0.172)	0.099 (0.136)	<0.001*
**3076**	2511 (0.270)	1595 (0.146)	<0.001*	0.042 (0.094)	0.020 (0.065)	<0.001*
**2879**	2236 (0.241)	1103 (0.101)	<0.001*	0.052 (0.137)	0.008 (0.032)	<0.001*
**2980**	5695 (0.613)	6870 (0.629)	<0.001*	0.091 (0.107)	0.081 (0.089)	<0.001*
**2636**	6447 (0.693)	8076 (0.740)	<0.001*	0.250 (0.293)	0.315 (0.318)	<0.001*
**6593**	5213 (0.561)	4032 (0.369)	<0.001*	0.052 (0.061)	0.040 (0.067)	<0.001*
**2306**	6606 (0.711)	8013 (0.734)	<0.001*	0.297 (0.337)	0.326 (0.330)	<0.001*
**2910**	1412 (0.152)	357 (0.033)	<0.001*	0.016 (0.052)	0.003 (0.018)	<0.001*
**3045**	3366 (0.362)	4414 (0.404)	<0.001*	0.069 (0.130)	0.064 (0.113)	0.002
**2287**	6944 (0.747)	8269 (0.758)	<0.001*	0.283 (0.311)	0.304 (0.310)	<0.001*
**2200**	5530 (0.595)	6191 (0.567)	<0.001*	0.233 (0.308)	0.243 (0.320)	0.021
**2547**	4421 (0.476)	3560 (0.326)	<0.001*	0.079 (0.126)	0.041 (0.080)	<0.001*

**6569**	227 (0.024)	1381 (0.127)	<0.001*	0.002 (0.018)	0.015 (0.045)	<0.001*
**3056**	8689 (0.935)	10165 (0.931)	<0.001*	0.187 (0.141)	0.168 (0.127)	<0.001*
**6614**	8263 (0.889)	8393 (0.769)	<0.001*	0.072 (0.046)	0.068 (0.052)	<0.001*
**15154**	4911 (0.528)	7238 (0.663)	<0.001*	0.010 (0.017)	0.015 (0.021)	<0.001*
**5033**	8413 (0.905)	9022 (0.827)	<0.001*	0.169 (0.118)	0.161 (0.123)	<0.001*
**2069**	7784 (0.837)	8735 (0.800)	<0.001*	0.228 (0.249)	0.195 (0.224)	<0.001*
**3277**	2931 (0.315)	3119 (0.286)	0.016	0.031 (0.054)	0.025 (0.047)	<0.001*
**2397**	1272 (0.137)	133 (0.012)	<0.001*	0.019 (0.064)	0.002 (0.022)	<0.001*
**6424**	9203 (0.990)	10597 (0.971)	<0.001*	0.281 (0.160)	0.278 (0.172)	0.153
**6890**	9143 (0.983)	10428 (0.955)	<0.001*	0.558 (0.322)	0.563 (0.340)	0.228
**7527**	7846 (0.844)	9541 (0.874)	<0.001*	0.077 (0.082)	0.097 (0.097)	<0.001*
**6438**	213 (0.023)	1612 (0.148)	<0.001*	0.003 (0.030)	0.042 (0.121)	<0.001*
**2966**	7585 (0.816)	9310 (0.853)	<0.001*	0.085 (0.073)	0.084 (0.067)	0.212
**9629**	9033 (0.972)	10590 (0.970)	<0.001*	0.335 (0.195)	0.362 (0.206)	<0.001*
**7021**	6021 (0.648)	7257 (0.665)	<0.001*	0.055 (0.053)	0.061 (0.056)	<0.001*
**7568**	9162 (0.985)	10762 (0.986)	<0.001*	0.211 (0.121)	0.238 (0.125)	<0.001*
**3765**	235 (0.025)	1328 (0.122)	<0.001*	0.002 (0.022)	0.009 (0.034)	<0.001*
**3445**	8865 (0.954)	10526 (0.964)	<0.001*	0.235 (0.169)	0.253 (0.171)	<0.001*

Supplementary Table 7. Results of Chi-square tests and t-tests for selected features on erythromycin model.

	Number (Proportion) of spectra			Mean (Standard devation) of intensity		
M/Z	R	S	p-value	R	S	p-value
**6593**	6531 (0.578)	2714 (0.305)	0	0.059 (0.068)	0.028 (0.056)	<0.001*
**2413**	3189 (0.282)	238 (0.027)	0	0.131 (0.272)	0.004 (0.031)	0
**5526**	10579 (0.936)	6510 (0.731)	<0.001*	0.336 (0.225)	0.270 (0.240)	<0.001*
**2432**	2953 (0.261)	268 (0.030)	0	0.059 (0.142)	0.005 (0.033)	<0.001*
**6553**	9142 (0.809)	7056 (0.792)	<0.001*	0.123 (0.104)	0.121 (0.093)	0.237
**3040**	8870 (0.785)	6489 (0.728)	<0.001*	0.320 (0.335)	0.245 (0.293)	<0.001*
**6568**	240 (0.021)	1243 (0.140)	<0.001*	0.002 (0.020)	0.016 (0.046)	<0.001*
**3008**	9775 (0.865)	7578 (0.851)	<0.001*	0.451 (0.394)	0.403 (0.374)	<0.001*
**2456**	2504 (0.222)	295 (0.033)	0	0.041 (0.108)	0.005 (0.033)	<0.001*
**7595**	3198 (0.283)	1041 (0.117)	<0.001*	0.020 (0.041)	0.008 (0.027)	<0.001*
**3029**	5966 (0.528)	4591 (0.515)	<0.001*	0.114 (0.162)	0.095 (0.136)	<0.001*
**3068**	5349 (0.473)	3454 (0.388)	<0.001*	0.082 (0.139)	0.059 (0.114)	<0.001*
**2980**	7295 (0.645)	5270 (0.592)	<0.001*	0.092 (0.101)	0.076 (0.089)	<0.001*
**6614**	9986 (0.883)	6670 (0.749)	<0.001*	0.072 (0.045)	0.067 (0.053)	<0.001*
**6438**	263 (0.023)	1562 (0.175)	<0.001*	0.003 (0.030)	0.051 (0.131)	<0.001*
**5033**	10253 (0.907)	7182 (0.806)	<0.001*	0.170 (0.116)	0.158 (0.126)	<0.001*
**6905**	791 (0.070)	1861 (0.209)	<0.001*	0.011 (0.067)	0.101 (0.249)	<0.001*
**2636**	8026 (0.710)	6503 (0.730)	<0.001*	0.264 (0.297)	0.311 (0.318)	<0.001*
**2547**	5155 (0.456)	2849 (0.320)	<0.001*	0.072 (0.118)	0.040 (0.079)	<0.001*
**2306**	8228 (0.728)	6391 (0.717)	<0.001*	0.307 (0.335)	0.319 (0.330)	0.010

**6424**	11188 (0.990)	8612 (0.967)	<0.001*	0.287 (0.163)	0.268 (0.171)	<0.001*
**3056**	10559 (0.934)	8336 (0.936)	<0.001*	0.180 (0.136)	0.175 (0.129)	0.009
**6524**	281 (0.025)	1442 (0.162)	<0.001*	0.002 (0.015)	0.023 (0.059)	<0.001*
**6890**	11103 (0.982)	8399 (0.943)	<0.001*	0.573 (0.322)	0.540 (0.344)	<0.001*
**5290**	6377 (0.564)	5341 (0.600)	<0.001*	0.057 (0.068)	0.092 (0.127)	<0.001*
**13128**	163 (0.014)	775 (0.087)	<0.001*	0.000 (0.004)	0.002 (0.008)	<0.001*
**4514**	1411 (0.125)	1985 (0.223)	<0.001*	0.011 (0.038)	0.023 (0.054)	<0.001*
**2287**	8563 (0.758)	6650 (0.747)	<0.001*	0.290 (0.310)	0.297 (0.309)	0.106
**7527**	10001 (0.885)	8089 (0.908)	<0.001*	0.092 (0.095)	0.111 (0.110)	<0.001*
**6928**	9494 (0.840)	6517 (0.732)	<0.001*	0.103 (0.085)	0.096 (0.089)	<0.001*
**3445**	10825 (0.958)	8566 (0.962)	<0.001*	0.238 (0.169)	0.254 (0.172)	<0.001*
**9629**	10988 (0.972)	8635 (0.969)	<0.001*	0.341 (0.199)	0.358 (0.205)	<0.001*
**5004**	62 (0.005)	1246 (0.140)	<0.001*	0.001 (0.014)	0.024 (0.070)	<0.001*
**2652**	10987 (0.972)	8721 (0.979)	<0.001*	0.253 (0.167)	0.279 (0.177)	<0.001*
**2200**	6904 (0.611)	4817 (0.541)	<0.001*	0.245 (0.313)	0.229 (0.315)	<0.001*
**7021**	7581 (0.671)	5697 (0.640)	<0.001*	0.058 (0.054)	0.058 (0.056)	0.469
**2069**	9301 (0.823)	7218 (0.810)	<0.001*	0.216 (0.243)	0.201 (0.227)	<0.001*

Supplementary Table 8. Number of data in training set and independent test set for constructing multiple antibiotics resistance model.

	**Training set**	**Independent test set**
**Resistant to three antibiotics**	8234	1590
**Susceptible to three antibiotics**	7455	1964
**Total**	15689	3554

Supplementary Table 9. Results of 10-fold cross validation with the optimal parameters based on the grid search

bw_method	#Features	Model	SN	SP	ACC	MCC	AUC
0.0014	805	DT	0.8430	0.8179	0.8312	0.6614	0.8304
		RF	0.8573	0.9138	0.8843	0.7704	0.8856
		SVM	0.8615	0.8738	0.8676	0.7350	0.8677
0.0012	910	DT	0.8445	0.8318	0.8386	0.6762	0.8382
		RF	0.8686	0.9129	0.8898	0.7807	0.8908
		SVM	0.8713	0.8803	0.8758	0.7512	0.8758
0.001	1053	DT	0.8558	0.8335	0.8452	0.6895	0.8446
		RF	0.8732	0.9150	0.8932	0.7874	0.8941
		SVM	0.8741	0.8842	0.8790	0.7578	0.8791
0.0008	1223	DT	0.8573	0.8386	0.8484	0.6960	0.8480
		RF	0.8760	0.9121	0.8933	0.7873	0.8940
		SVM	0.8776	0.8840	0.8807	0.7611	0.8808
0.0006	1461	DT	0.8550	0.8322	0.8442	0.6875	0.8436
		RF	0.8799	0.9118	0.8952	0.7909	0.8958
		SVM	0.8784	0.8897	0.8839	0.7675	0.8840
